# Potential of Individual Upper-Limb Muscles to Contribute to Postural Tremor: Simulations From Neural Drive to Joint Rotation

**DOI:** 10.5334/tohm.949

**Published:** 2025-02-25

**Authors:** Spencer A. Baker, Landon J. Beutler, Daniel B. Free, Dario Farina, Steven K. Charles

**Affiliations:** 1 Mechanical Engineering, Brigham Young University, Provo, Utah, US; 2 Bioengineering, Imperial College London, London, United Kingdom; 3 Neuroscience, Brigham Young University, Provo, Utah, US

**Keywords:** Tremor, propagation, muscle, tremorogenic, model, simulation

## Abstract

**Background:**

It is unclear which muscles contribute most to tremor and should therefore be targeted by tremor suppression methods. Previous studies used mathematical models to investigate how upper-limb biomechanics affect muscles’ potential to generate tremor. These investigations yielded principles, but the models included at most only 15 muscles. Here we expand previous models to include 50 upper-limb muscles, simulate tremor propagation, and test the validity of the previously postulated principles.

**Methods:**

Tremor propagation was characterized using the gains between tremorogenic neural drive to the 50 muscles (inputs) and tremulous joint rotations in the 7 joint degrees-of-freedom (DOF) from shoulder to wrist (outputs). Each gain can be interpreted as the potential of a muscle to generate tremor in a DOF. Robustness and sensitivity analyses were performed to assess the effects of model parameter variability on gains.

**Results:**

Simulations of postural tremor using the expanded model confirmed the previously postulated principles and revealed new insights, including: 1) most of the muscles with the largest gains were among the 15 muscles in the original model; 2) some gains depended strongly on posture; 3) averaged across the postures included in this study, the largest gains belonged to input-output pairs involving biceps/forearm/wrist muscles and forearm/wrist DOF, 4) although some shoulder and extrinsic hand muscles also exhibited large gains, especially in select postures.

**Discussion:**

These observations suggest that in the absence of additional information (such as tremorogenic neural drive to muscles), peripheral tremor suppression efforts should start by targeting biceps/forearm/wrist muscles or forearm/wrist DOF.

## 1 Introduction

Tremor, defined as an involuntary, rhythmic movement of one or multiple body parts [1], is one of the most common movement disorders [2]. It occurs in a variety of diseases, including Essential Tremor, Parkinson’s disease, dystonia, cerebellar ataxia, corticobasal degeneration, Leigh’s syndrome, and multiple system atrophy [3]. Essential Tremor alone affects approximately seven million people in the United States [4].

Many persons with tremor find the most common treatment options—medication, deep brain stimulation, or focused ultrasound thalamotomy—unsatisfactory. Medication is effective in only 50% of patients and reduces tremor in these patients on average by only 50% [1,5,6]; in addition, medication frequently causes undesirable side effects [7]. Deep brain stimulation and focused ultrasound thalamotomy can be very effective but carry the risk and intimidation associated with neurosurgery. For example, deep brain stimulation reduces tremor by approximately 90% [1,6], but due in part to its invasive nature, side effects [8,9,10,11,12], and potential loss of efficacy [13], only about 3% of tremor patients receive this treatment [14]. Consequently, many tremor patients desire alternative solutions [15].

Other treatments have shown potential but have not yet been optimized. Alternatives to the common treatment options attempt to intervene peripherally, for example using low-pass filtering orthoses or robotic exoskeletons [16,17,18,19,20,21,22,23], tremor-canceling activation of antagonist muscles [24,25,26,27], botulinum toxin [28,29,30,31], or submotor-threshold electrical stimulation [24,32,33,34,35,36,37]. However, we do not currently know which muscles are most responsible for tremor, making it difficult to know where (which muscles/joints) to intervene to maximize tremor suppression while minimizing undesirable side effects.

Determining which muscles are most responsible for tremor is a complex challenge [28]. Simulations have shown that because tremor propagates along the upper limb, tremorogenic activity in a single muscle can cause tremor in most or all joint degrees of freedom (DOF), including DOF that are far from that muscle [38]. Upper-limb tremor represents a convolution of tremorogenic activity in all upper-limb muscles, making it difficult to determine which muscles cause the most tremor and should therefore be targeted.

To investigate the progression from tremorogenic neural drive to tremor, we recently developed a model of tremor propagation [38]. This model transformed neural drive to the 15 major superficial muscles of the upper limb (inputs) into joint rotation in the 7 major joint DOF from the shoulder to the wrist (outputs). From simulations using this model, we postulated six principles underlying tremor propagation: 1) The distribution of tremor (among the joint DOF of the upper limb) depends strongly on musculoskeletal dynamics, not just on which muscles have the most tremorogenic activity. 2) Tremor spreads (from tremorogenic drive to a given muscle to joint DOF throughout the upper limb) primarily because of inertial interaction torques and multi-articular muscles; interaction torques due to joint stiffness and damping also spread tremor, but less than inertial interaction torques. 3) Tremor spreads narrowly, but not necessarily locally: although tremorogenic activity in a given muscle creates tremor in most or all DOF, most of the tremor caused by a given muscle occurs in a small number of DOF, which are not necessarily close to the muscle. 4) Assuming that all muscles receive the same amount of tremorogenic activity, tremor increases proximal-distally, and most of the tremor in distal DOF comes from muscles that act directly on those DOF. 5) Although inertial weighting is sometimes used to suppress tremor, increasing inertia can increase tremor in some cases. 6) Similarly, increasing joint viscoelasticity decreases tremor in most cases but can increase tremor in some cases [38].

This model has provided an important starting point for elucidating tremor propagation, but it suffers from a major shortcoming: the model included input (tremorogenic neural drive) to only 15 of the 50 muscles from shoulder to wrist. Therefore, the purpose of this study was to expand the model to include activity in an additional 35 muscles as inputs, and to use this expanded model to simulate tremor propagation and test the validity of (and update, if necessary) the previously postulated principles of tremor propagation. Since the upper-limb musculoskeletal system does not depend strongly on the tremorogenic neural drive to muscles, this model and the associated principles apply to all types of upper-limb tremor, whether pathological or physiological in nature.

## 2 Methods

### 2.1 Model

**Figure 1:**
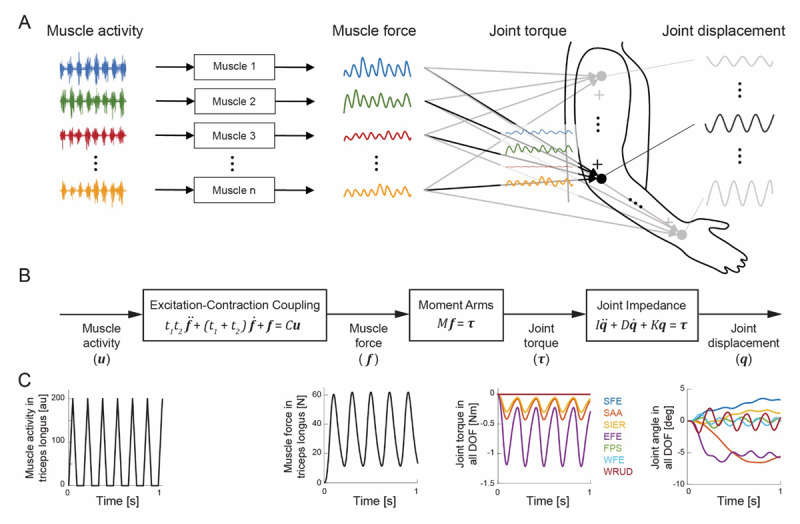
Tremor propagation. **A.** Overview: tremorogenic activity in multiple muscles generates tremorogenic forces in the same muscles before combining into tremorogenic torques and finally tremor in joint DOF throughout the upper limb. **B.** Model: this process is modeled using three sub-models that transform activity in 50 muscles (***u***) into force in the same muscles (***f***), then joint torque (*τ*) and finally joint rotation (***q***) in the 7 joint DOF from shoulder to wrist. Variables ***u*** and ***f*** are 50-element time-varying vectors, and *τ* and ***q*** are 7-element time-varying vectors. Parameters *t*1, *t*2, and *C* are 50-by-50 diagonal matrices representing time constants of excitation-contraction coupling (*t*1 and *t*2) and maximum muscle force (*C*). Parameter *M* is a 7-by-50 matrix of muscle moment arms, and *I, D*, and *K* are 7-by-7 impedance matrices representing coupled joint inertia, damping, and stiffness, respectively. **C.** Example: simulated tremorogenic activity in the triceps longus muscle generates tremorogenic force in the triceps longus muscle, tremorogenic torque in the DOF of the shoulder and elbow, and finally tremor in all 7 DOF. These simulations include both the transient and steady-state responses, but the rest of the paper considers only steady-state responses.

In this study, we focused on postural tremor. In previous investigations of postural tremor [38,39], we modeled the upper limb as a linear, time-invariant, multi-input multi-output system (Figure 1A-B), with tremorogenic neural drive to various muscles as inputs and tremulous joint rotations in the joint DOF of the upper limb as outputs. Because postural tremor involves relatively small deviations^1^ about an equilibrium posture and does not necessarily involve large changes in neuromusculoskeletal properties while maintaining a given posture, it is well-suited to be approximated as a linear, time-invariant system; therefore, we continued using this model structure to maintain tractability and focus on the most fundamental principles of tremor propagation.

**Figure 2:**
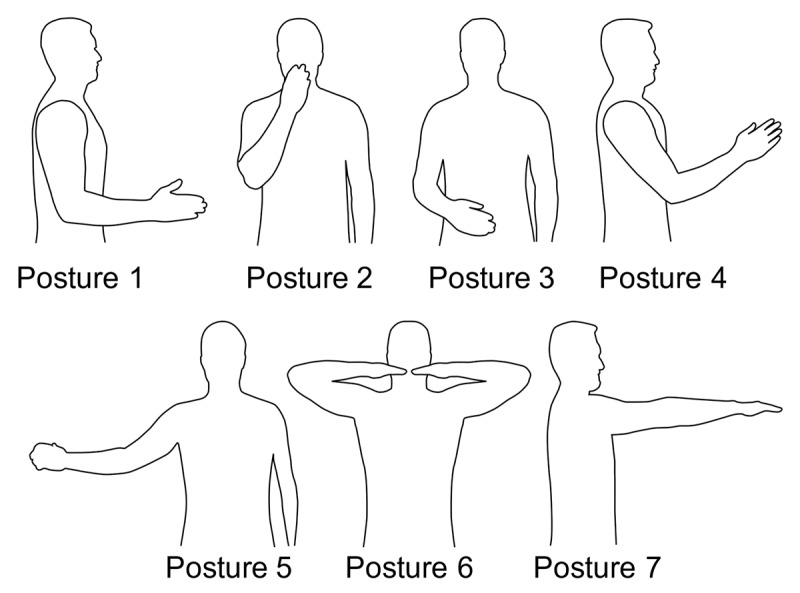
Tremor propagation was simulated in these seven postures. Postures 1–5 are representative of activities of daily living: anatomical posture, but with the elbow flexed at 90° and the forearm midway between pronation and supination (Posture 1); hand held in front of the mouth (Posture 2); hand held in front of the abdomen (Posture 3); upper limb partially extended in front of the torso (Posture 4); and upper limb partially extended to the side and in front of the torso, roughly midway between the parasagittal and frontal planes (Posture 5). Postures 6 and 7 are common in clinical evaluation of tremor using the TETRAS [75]: wing beating posture (Posture 6) and forward horizontal reach posture (Posture 7). For exact joint angles, see Table 2.

Because tremor depends on limb configuration, we simulated tremor propagation in seven different postures (Figure 2). The first five postures broadly represented activities of daily living: maintaining the upper limb in the para-sagittal plane, with the hand forward (Posture 1); holding the hand in front of the mouth (Posture 2) or abdomen (Posture 3); and partially extending the upper limb in front of the torso (Posture 4) or midway between the parasagittal and coronal planes (Posture 5). The final two postures are used clinically to assess tremor: assuming the lateral wing beating posture (Posture 6) or forward horizontal reach posture (Posture 7). Joint angles in each posture are listed in Table 2.

#### 2.1.1 Inputs

The inputs to the expanded model consisted of tremorogenic neural drive to the 50 muscles of the upper limb acting on the 7 DOF from shoulder to wrist (Table 1). This set of 50 muscles included extrinsic hand muscles but excluded intrinsic hand muscles, which have no moment arm—and therefore cannot directly produce torque—across the 7 DOF included in the model (see 4.2 Limitations). Neural drive was expressed as a fraction of voluntary maximum contraction. This neural drive has been measured as surface electromyography (sEMG) in patients with tremor. Such sEMG recordings show bursts of activity repeated at the tremor frequency, which generally falls within the 4–8 Hz “tremor band” [3]. The rectified bursts can be approximated as triangles [40,41] with a mean width of 110 ms [42] and input into the tremor propagation model to simulate tremor output, as shown in Figure 1C. However, because the neuromusculoskeletal system heavily low-pass filters the signal on its way from input to output, the exact shape of the repeating waveform in the input has virtually no effect on the steady-state shape of the output, which is almost purely sinusoidal [38]. In other words, the neuromusculoskeletal system low-pass filters higher-frequency content in the input, leaving almost only content at the fundamental frequency (i.e. tremor frequency). Therefore, the tremorogenic neural drive to muscle can be approximated as a sinusoid at the tremor frequency, with virtually no change to the simulated steady-state tremor output.

**Table 1:** List of muscles included in the model, together with abbreviations and peak forces, ordered roughly from proximal to distal.


MUSCLE	ABBREVIATION	PEAK FORCE (N)

Deltoid Anterior	DELT1	1218.9

Deltoid Middle	DELT2	1103.5

Deltoid Posterior	DELT3	201.6

Supraspinatus	SUPSP	499.2

Infraspinatus	INFSP	1075.8

Subscapularis	SUBSC	1306.9

Teres minor	TMIN	269.5

Teres major	TMAJ	144.0

Pectoralis major Clavicular	PECM1	444.3

Pectoralis major Sternal	PECM2	658.3

Pectoralis major Ribs	PECM3	498.1

Latissimus dorsi Thoracic	LAT1	290.5

Latissimus dorsi Lumbar	LAT2	317.5

Latissimus dorsi Iliac	LAT3	189.0

Coracobrachialis	CORB	208.2

Triceps Long	TRIlong	771.8

Triceps Lateral	TRIlat	717.5

Triceps Medial	TRImed	717.5

Anconeus	ANC	283.2

Supinator	SUP	379.6

Biceps Long	BIClong	525.1

Biceps Short	BICshort	316.8

Brachialis	BRA	1177.4

Brachioradialis	BRD	276.0

Extensor carpi radialis longus	ECRL	337.3

Extensor carpi radialis brevis	ECRB	252.5

Extensor carpi ulnaris	ECU	192.9

Flexor Carpi radialis	FCR	407.9

Flexor capri ulnaris	FCU	479.8

Palmaris longus	PL	101.0

Pronator teres	PT	557.2

Pronator quadratus	PQ	284.7

Flexor digitorum superficialis Digit 5	FDSL	75.3

Flexor digitorum superficialis Digit 4	FDSR	171.2

Flexor digitorum superficialis Digit 3	FDSM	258.8

Flexor digitorum superficialis Digit 2	FDSI	162.5

Flexor digitorum produndus Digit 5	FDPL	236.8

Flexor digitorum produndus Digit 4	FDPR	172.9

Flexor digitorum produndus Digit 3	FDPM	212.4

Flexor digitorum produndus Digit 2	FDPI	197.3

Extensor digitorum communis Digit 5	EDCL	39.4

Extensor digitorum communis Digit 4	EDCR	109.2

Extensor digitorum communis Digit 3	EDCM	94.4

Extensor digitorum communis Digit 2	EDCI	48.8

Extensor digiti minimi	EDM	72.4

Extensor indicis propius	EIP	47.3

Extensor pollicis longus	EPL	88.3

Extensor pollicis brevis	EPB	46.0

Flexor pollicis longus	FPL	201.0

Abductor pollicis longus	APL	116.7


**Table 2:** Joint angles for the 7 postures considered in simulation, listed according to the Denavit-Hartenberg parameterization set forth in [39]. The postures are meant to reflect a range of activities of daily living (1–5) in addition to postures commonly used for clinical diagnosis (6–7). See Figure 2 for a description and visualization of each posture.


	POSTURE 1	POSTURE 2	POSTURE 3	POSTURE 4	POSTURE 5	POSTURE 6	POSTURE 7

*θ_1_*	0	*π*/4	*π*/16	*π*/5	*π*/3	*π*/2	*π*/2

*θ_2_*	0	0	*–π*/16	*π*/8	*–π*/3	*–5π*/18	*0*

*θ_3_*	0	*π*/4	*π*/3	*π*/8	*π*/6	*5π*/12	*π*/2

*θ_4_*	*π*/2	*3π*/4	*π*/2	*π*/3	*π*/6	*25π*/36	*0*

*θ_5_*	*π*/2	*π*/4	*π*/2	*π*/4	*π*/4	*7π*/16	*π*/2

*θ_6_*	0	0	0	0	0	0	0

*θ_7_*	0	0	0	0	0	0	0


#### 2.1.2 Outputs

The model outputs were joint rotations in the seven major joint DOF of the upper limb: shoulder flexion/extension (SFE), adduction/abduction (SAA), and internal/external rotation (SIER); elbow flexion/extension (EFE); forearm pronation/supination (FPS); and wrist flexion/extension (WFE) and radial/ulnar deviation (WRUD).

#### 2.1.3 Submodels

The upper-limb model is comprised of three sub-models (Figure 1B). These sub-models and their parameters were described in detail previously [38], so we provide only a summary here.

*Muscle activation to muscle force:* The first sub-model, which represents muscle excitation-contraction dynamics, converts muscle activation to muscle force (Figure 1B). It has 50 inputs (normalized neural drive in each muscle) and 50 outputs (force in each muscle). Activation in a muscle produces force in the same muscle and does not affect the other muscles. The excitation-contraction dynamics of each muscle were modeled as a linear Hill-type muscle model [43], resulting in a slightly over-damped second-order system with time constants (*t*_1_ and *t*_2_) and maximum voluntary force (*C*). Default values for these parameters were taken from prior studies. Specifically, since upper-limb muscles appear to exhibit very similar time constants [44,45,46], all muscles were assumed to have the same time constants (*t*_1_ = 30 ms and *t*_2_ = 40 ms) but different maximum voluntary force values [47,48,49], listed in Table 1. The effect of other time constant values is explored in the robustness analysis (details below).

*Muscle force to joint torque:* The second sub-model in Figure 1B represents the musculoskeletal geometry of the upper limb, through which muscle forces create joint torques via moment arms. The sub-model inputs are the 50 muscle forces from the first sub-model, and the outputs are the resulting joint torques in the 7 DOF. In contrast to the first sub-model, an input in a single muscle produces outputs in multiple DOF. The moment-arm matrix (*M*) includes the moment arms of all muscles onto all 7 DOF. Moment arm values were obtained from an OpenSim model [47,50] with height and mass (Table SM3, taken from [51]) and body-segment proportions (Table SM4, taken from [52]) scaled to represent the average between a 50^th^ percentile female and a 50^th^ percentile male. Since moment arm values depend on limb configuration, we obtained moment arms specific to each of the seven postures shown in Figure 2 (Tables SM5–SM11 in Supplemental Material).

*Joint torque to joint rotation:* The third sub-model represents the mechanical joint impedance of the upper limb (i.e. the combined effects of upper-limb inertia, damping, and stiffness, expressed in joint space). The inputs are the torques in the seven DOF produced by the second sub-model. The outputs are the joint rotations in the same seven DOF. The DOF in the fingers were not included in the model (see 4.2 Limitations). The inertia, damping, and stiffness matrices (*I, D*, and *K*) include the off-diagonal (coupling) terms that result in interaction torques; therefore, a torque in a single DOF can produce joint rotation in multiple DOF. Importantly, the damping and stiffness matrix values were gathered from past experimental measurements in which joints were perturbed (e.g. by an applied torque) and the resulting responses were recorded; therefore, they include the basic (i.e. linear) viscoelastic effects of all perturbed tissues, including bone, joint, and muscle.^2^ In these experiments, the perturbed joints were not at or close to the ends of their ranges of motion; similarly, in the postures simulated in this study (Figure 2), the joints were not at or close to the ends of their ranges of motion (with one exception—see below). Default values for the damping and stiffness matrices (Tables SM1 and SM2 in Supplemental Material) were taken from [38,53,54,55]; for full details, see [39]. The exception is Posture 7, in which the elbow was fully extended; to reflect the dramatic increase in viscoelasticity of the locked elbow, we greatly increased elbow joint stiffness and damping in this posture (by factors of 10,000 and 100, respectively). Using the Robotics, Vision, and Control toolbox [56], inertia matrix values were calculated from the height and mass (Table SM3, taken from [51]) and body-segment proportions (Table SM4, taken from [52]) representing the average between a 50^th^ percentile female and a 50^th^ percentile male. Similar to the moment arm matrix, the inertia matrix depends on limb configuration, so we calculated the inertia matrix in each posture.

#### 2.1.4 Gain

The approximation of tremorogenic neural drive to muscle as sinusoidal (see 2.1.1) allowed us to quantify steady-state tremor propagation as the frequency response of the neuromusculoskeletal system at the tremor frequency [57]. With 50 inputs and 7 outputs, the system has 350 input-output relationships (transfer functions), and the frequency response of each input-output relationship is characterized by its gain (aka magnitude ratio) and phase shift at the tremor frequency. The gain of a given input-output relationship is the amount by which the system amplifies (or reduces, if the gain is less than 1) an input on its way to becoming an output. For example, if the gain between tremorogenic neural drive to a given muscle (expressed as a fraction of maximum voluntary contraction, MVC) and tremor in a given DOF (expressed in radians) is 2 rad/MVC, then tremorogenic neural drive of amplitude 0.1 MVC to that muscle would produce tremor of amplitude 0.2 rad in that DOF. Importantly, the gains between inputs and outputs depend only on the system and not on the amplitudes or phases of the inputs, which, in the case of tremorogenic neural drive to muscles, can fluctuate substantially within a subject, even over short time periods [58]. Therefore, instead of describing tremor propagation in terms of the tremor that results from certain combinations of input amplitudes and phases, we present tremor propagation in terms of gains of input-output pairs. Each gain can be interpreted as the potential of tremorogenic neural drive to a given muscle to generate tremor in a given DOF.

### 2.2 Analysis

As stated above, the purpose of expanding the model was to test the validity of the previously postulated principles when the tremor propagation model includes inputs to all 50 muscles (instead of 15). To this end, we 1) simulated tremor propagation with default model parameter values, 2) performed a robustness analysis to assess the stability of these patterns to physiologically plausible variations in model parameter values, and 3) performed a sensitivity analysis to identify the effect of increasing or decreasing inertia, damping, and stiffness on tremor.

#### 2.2.1 Tremor Simulations

As mentioned above, the increase in signal amplitude from model input to model output is captured by the gains of the system. The same is true for each sub-model, so we calculated the gains of each sub-model from its transfer functions, evaluated at the tremor frequency (for details, see Corie et al [38]). This allowed us to simulate the effect of the musculoskeletal system on intermediate variables (muscle forces and joint torques) in addition to the final outputs (joint rotations).

#### 2.2.2 Robustness Analysis

Our model parameter values were carefully gathered from the literature and represent average measured values. Nonetheless, there is uncertainty in the average parameter values, not to mention variation in the values within and between subjects. Therefore, we performed an analysis to determine the robustness of the default simulation results to physiologically plausible variations in the following parameters. To quantify the amount of variation in gains caused by varying parameters, we calculated correlation coefficients for all pairwise comparisons.

*Tremor frequency:* To include a variety of tremor frequencies, we calculated the gains throughout the 4–8 Hz tremor band. To quantify the amount of similarity/difference between gains at different integer tremor frequencies (4, 5, 6, 7, and 8 Hz), we calculated correlation coefficients for all pairwise comparisons (e.g. gains at 4 vs 5 Hz, 4 vs 6 Hz, etc.).

*Muscle dynamics:* Simulations were repeated with the muscle time constants set at 0.5 and 2 times their default value, which surpasses the range of the time constants found in upper body muscles [46]. We also tested the effect of not assuming that all muscles had the same dynamics by randomly choosing *t*_1_ and *t*_2_ (separately for each muscle) from normal distributions centered on the default values (30 and 40ms), with standard deviations of 3 and 4ms, respectively. These standard deviations correspond to a coefficient of variation of 10%, which is roughly what has been observed in upper-limb muscles [46].

*Maximum muscle force:* The gain between a given muscle and DOF is proportional to the maximum muscle force of that muscle (*C*). If this maximum force is increased/decreased, the gain would increase/decrease by the same ratio. Therefore, no robustness analysis of this parameter was necessary.

*Subject size:* The size of an individual affects the moment-arm and inertia matrices. As mentioned above, the default model incorporated parameters averaged from a 50^th^ percentile male and female. We repeated the simulations with models representing 10^th^ and 50^th^ percentile females and 50^th^ and 90^th^ percentile males. Moment-arm values were again obtained from an OpenSim model [47,50], scaled according to subject height and mass (Table SM3, taken from [51]) and body-segment proportions (Table SM4, taken from [52]). Inertial values were also adjusted based on these estimates.

*Co-contraction:* The default model parameter values assumed no co-contraction. Muscle co-contraction leads to an increase in both joint stiffness and joint damping [59]; joint stiffness increases roughly proportionally to the subject’s voluntary torque, and joint damping increases roughly by the square-root of that factor [60]. To evaluate how tremor propagation is affected by co-contraction, we multiplied the stiffness matrix by factors of 0.5, 1, 2, 4, and 8, and the damping matrix by the square root of those factors. This range in stiffness roughly represents the range in wrist stiffness seen at various levels of co-contraction in previous studies [61,62,63.^64^,^65^,^66^].

#### 2.2.3 Sensitivity Analysis

Applying weight or viscoelastic bracing to the upper limb has been shown to decrease tremor in many cases [67,68,69]. We hypothesized that adding weight or viscoelastic bracing to the upper limb does not *always* decrease tremor and could, in some cases (depending on where the weight or bracing is applied), even increase tremor. To test this hypothesis, we performed a sensitivity analysis to identify the effect of slightly increasing joint inertia, damping, or stiffness (representing adding mass, viscosity, or elasticity, respectively) throughout the upper limb. Specifically, we numerically determined the normalized sensitivity of the gain (evaluated at the representative frequency of 6 Hz) of each of the 350 input-output relationships to each non-zero element of the inertia, damping, and stiffness matrices. The normalized sensitivity of gain *G_ij_* between input in muscle *j* and output in DOF *i* to parameter *p_k_* was defined as


\[
{S_{ijk}} = \frac{{\partial {G_{ij}}/{G_{ij}}}}{{\partial {p_k}/{p_k}}} = \frac{{\partial {G_{ij}}}}{{\partial {p_k}}}.\frac{{{p_k}}}{{{G_{ij}}}}
\]

where *K* refers any non-zero element of the inertia, damping, or stiffness matrices. As mentioned, we were particularly interested in observing whether increasing inertia, damping, and stiffness parameters could increase tremor. Therefore, in addition to computing the mean sensitivity of the gains of the 350 input-output relationships to each inertia, damping, and stiffness parameter, we also determined the percentage of input-output relationships that experienced an increase in gain when a given parameter was increased. Since Posture 7 was associated with abnormal stiffness and damping (see above), it was excluded from the sensitivity analysis for stiffness and damping. Note that this sensitivity analysis focuses on the effect of externally added weight or viscoelasticity in a given posture and cannot be extrapolated to predict the effect of altered joint inertia caused by changing posture (the latter effect is described separately in section 3.1.2).

## 3. Results

### 3.1 Tremor Simulations

#### 3.1.1 Individual stages of tremor propagation, averaged across all postures

**Figure 3:**
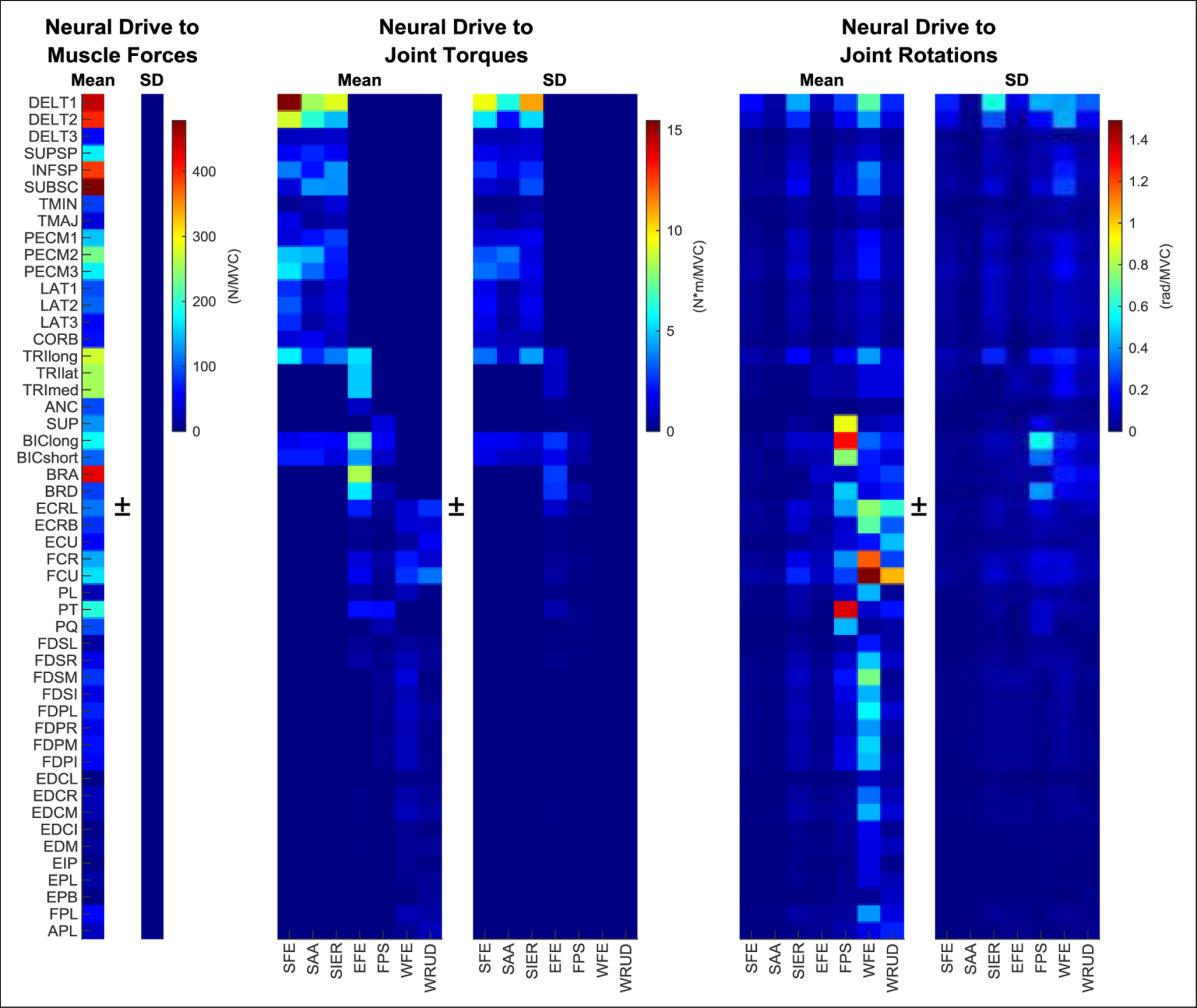
Gains from neural drive to muscle force, neural drive to joint torque, and neural drive to joint rotation, averaged across all postures (evaluated at a tremor frequency of 6 Hz). Alternatively, the heatmaps can be interpreted as the muscle force, joint torque, and joint rotation that would be result if all muscles received the same amount of tremorogenic activity (relative to each muscle’s maximum voluntary contraction). Muscles are ordered proximal (top) to distal (bottom; for muscle names, see Table 1). Similarly, joint DOF are listed proximal (left) to distal (right): shoulder flexion-extension (SFE), abduction-adduction (SAA), and internal-external rotation (SIER); elbow flexion-extension (EFE) and forearm pronation-supination (FPS); and wrist flexion-extension (WFE) and radial-ulnar deviation (WRUD), respectively. Equivalent heatmaps for individual postures are given in Figures SM1-SM7 in Supplemental Materials.

The model was used to evaluate tremor propagation at each stage (Figure 1), resulting in gains from neural drive to muscle force, joint torque, and joint rotations (Figure 3). After averaging across all postures included in this study, we noted the following trends in gains at each stage:^3^

*Neural drive to muscle force:* Unsurprisingly, because of the large maximum force of many proximal muscles (Table 1), the largest gains were associated with inputs to proximal muscles. By comparison, inputs to extrinsic hand muscles exhibited small gains.

*Neural drive to joint torque:* Since muscles have non-zero moment arms with respect to multiple DOF, force in a single muscle produced torque in multiple DOF. Not surprisingly, because of the large maximum forces and moment arms of many proximal muscles, the largest gains involved inputs into proximal muscles and outputs in proximal DOF.

*Neural drive to joint rotation*: In stark contrast to the transformations from neural drive to force and torque, the transformation from neural drive to joint rotation generally produced the largest gains in distal (forearm and wrist) DOF. Furthermore, the greatest gains in these distal DOF were associated with muscles that act directly on these DOF. Gains involving forearm pronation-supination were greatest from the biceps, supinator, and pronator teres muscles. For wrist flexion-extension, the primary wrist and extrinsic hand muscles produced the greatest gains. Similarly, the greatest gains in radial-ulnar deviation involved the primary wrist muscles. Thus, although some proximal muscles (e.g. anterior and middle deltoid, infraspinatus, and subscapularis muscles) produced their greatest gains in distal DOF (even though they do not cross these DOF), these gains were not as large as those produced by forearm and wrist muscles. For most of the muscles with large gains, one DOF clearly dominated over the others, even after averaging across all seven postures (ECRL and FCU are exceptions).

Though not a focus of this study, the heatmap of phase shifts in a single posture (Posture 1) is given in Figure SM9 of Supplemental Material.

#### 3.1.2 Effect of posture on tremor propagation

The effect of posture on tremor propagation is captured by the variation in gains between postures (standard deviation heatmaps in Figure 3).

*Neural drive to muscle force:* Because the model assumes that muscles’ maximum force-generating capacity is the same in different postures (see Limitations), the standard deviation in the gain (from neural drive to force) across postures was zero.

*Neural drive to joint torque:* Combining the large torques in proximal DOF with the large variety of proximal joint angles between postures (Figure 2), the standard deviation in the gains from neural drive to joint torque was largest for inputs to proximal muscles and outputs in proximal DOF. The anterior and middle deltoid and triceps longus muscles exhibited the largest variations. By comparison, gains involving distal muscles or distal DOF showed little variation.

*Neural drive to joint rotation*: Since joint impedance spreads tremor throughout the upper limb, it also spreads the variability across DOF. The largest effect of posture was seen in gains between the anterior and middle deltoid muscles and shoulder, forearm, and wrist DOF, as well as between biceps and brachioradialis muscles and forearm pronation-supination. Likely in part because the different postures did not involve large differences in distal joint angles (Figure 2), there was little variability in gains involving distal muscles.

Analyzing tremor propagation heatmaps in individual postures (Figures SM1-SM7 in Supplemental Material), we observed that whereas forearm and wrist muscles created significant tremor in all postures, shoulder/elbow muscles were associated with large gains in only some postures. These muscle-posture combinations included the anterior deltoid (Postures 2–6), middle deltoid (Posture 6), infraspinatus (Postures 1–4, 6), subscapularis (Posture 1), pectoralis major (Postures 2, 4), biceps longus (Postures 2, 4–5), and triceps longus muscles (Postures 3–6). Nevertheless, despite these differences, overall the gains from neural drive to joint rotation were somewhat stereotyped across postures: pairwise correlations across postures yielded mean ± SD correlation coefficients of 0.83 ± 0.081 (range 0.67–0.97).

### 3.2 Robustness Analysis

**Figure 4:**
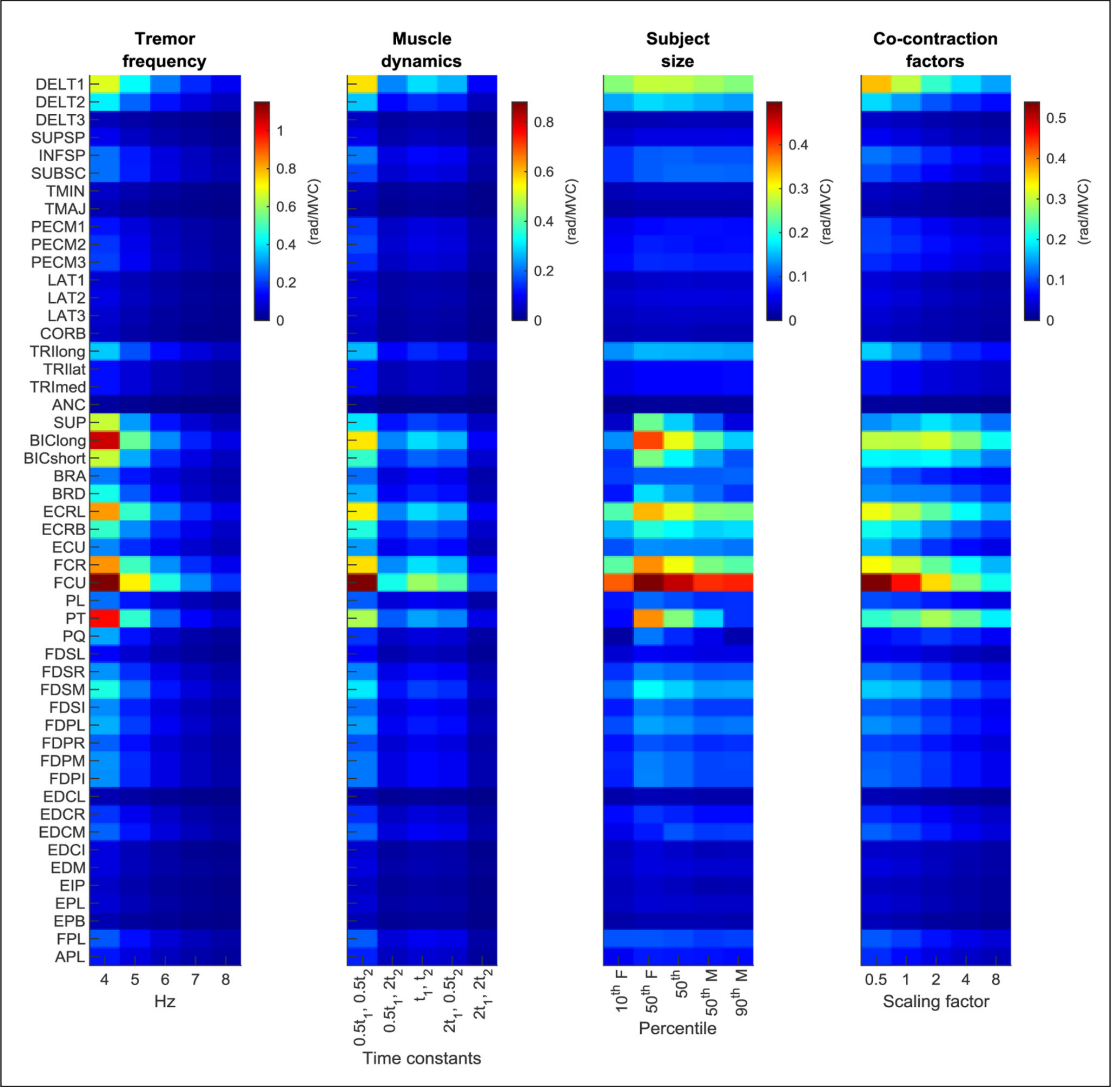
Tremor propagation patterns are quite robust to changes in tremor frequency, muscle dynamics, subject size, and co-contraction. Each cell represents the average of the gains from one muscle to joint rotation in all 7 DOF, averaged across all postures (i.e. the average of a row in the mean neural-drive-to-joint-rotation gain heatmap in Figure 3). Gains within a muscle (row) are generally more stereotyped than gains between muscles (column), demonstrated by the dominance of horizontal over vertical patterns.

To assess the stability of the above results to physiologically plausible variations in model parameter values, we performed a robustness analysis in each posture. To depict the results in a compact manner, we present the mean gain associated with each muscle (averaged across all seven DOF and all seven postures) for various model parameter values (Figure 4). Although changes in parameter values generally caused observable changes in gains, the relative gains (between muscles) were quite robust to changes in parameter values (with some exceptions noted below), as indicated by horizontal stripes in Figure 4.

*Tremor frequency:* We repeated the overall simulation (from neural drive to joint rotation) at tremor frequencies across the 4–8 Hz tremor band. The low-pass filtering behavior of the neuromusculoskeletal system caused gains to decrease at higher tremor frequencies, but the relative gains changed little with frequency (note the consistent pattern in the columns of the left-most plot in Figure 4). In other words, although tremor decreased at higher frequencies, the pattern of gains (how much tremor a given muscle can cause in a given DOF compared to other muscles and DOF) was quite stereotyped across tremor frequencies: pairwise correlations across tremor frequencies yielded correlation coefficients of 0.97 ± 0.033 (mean ± standard deviation; range 0.88–1.0).

*Muscle dynamics:* Shortening the time constants *t*_1_ and *t*_2_ caused an increase in gains across the tremor bandwidth, and lengthening the time constants caused a decrease in gains (Figure 4). However, because time constants were assumed to be the same for all muscles, increasing or decreasing time constants had no effect on the relative gains produced by each muscle compared to other muscles (i.e. R = 1). We also tested the effect of not assuming that all muscles had the same dynamics by varying the time constants randomly, both within and between muscles (not shown in Figure 4). We found that randomly selecting *t*_1_ and *t*_2_ from physiologically plausible distributions (see Methods) caused very little change in gain patterns (R = 0.99 ± 0.0033; range: 0.98–1.0).

*Subject size*: Overall, gains tended to decrease slightly with increasing subject size (Figure 4), likely caused by increased inertia outpacing the increase in moment arms. We did not increase maximum muscle force (tremor would simply scale proportionately), but an increase in muscle force with subject size would counter the observed decrease in gains. Either way, the change in gains with subject size was small compared to differences between muscles. Pairwise correlations across subject sizes yielded correlation coefficients of 0.90 ± 0.089 (range 0.68–.99).

*Co-contraction*: As voluntary co-contraction increased (approximated by a linear increase in joint stiffness and a square-rooted increase in damping), overall gain patterns changed little (R = 0.91 ± 0.078; range: 0.79–1.0). Gains associated with nearly all muscles decreased very slightly (Figure 4); a regression analysis showed that slopes of gain vs co-contraction factor were statistically significantly negative for 36% of gains (p ≤ 0.05), but the mean slope was small with a value of –0.0079 (rad/MVC)/(co-contraction scalar), where “co-contraction scalar” refers to the gain by which stiffness was multiplied (0.5, 1, 2, 4, 8). In other words, on average, doubling joint stiffness and increasing damping by a factor of \[
\sqrt 2
\] (compared to the default values) decreased the gain by only 0.0079 rad/MVC (0.45 deg/MVC).

### 3.3 Sensitivity Analysis

**Figure 5:**
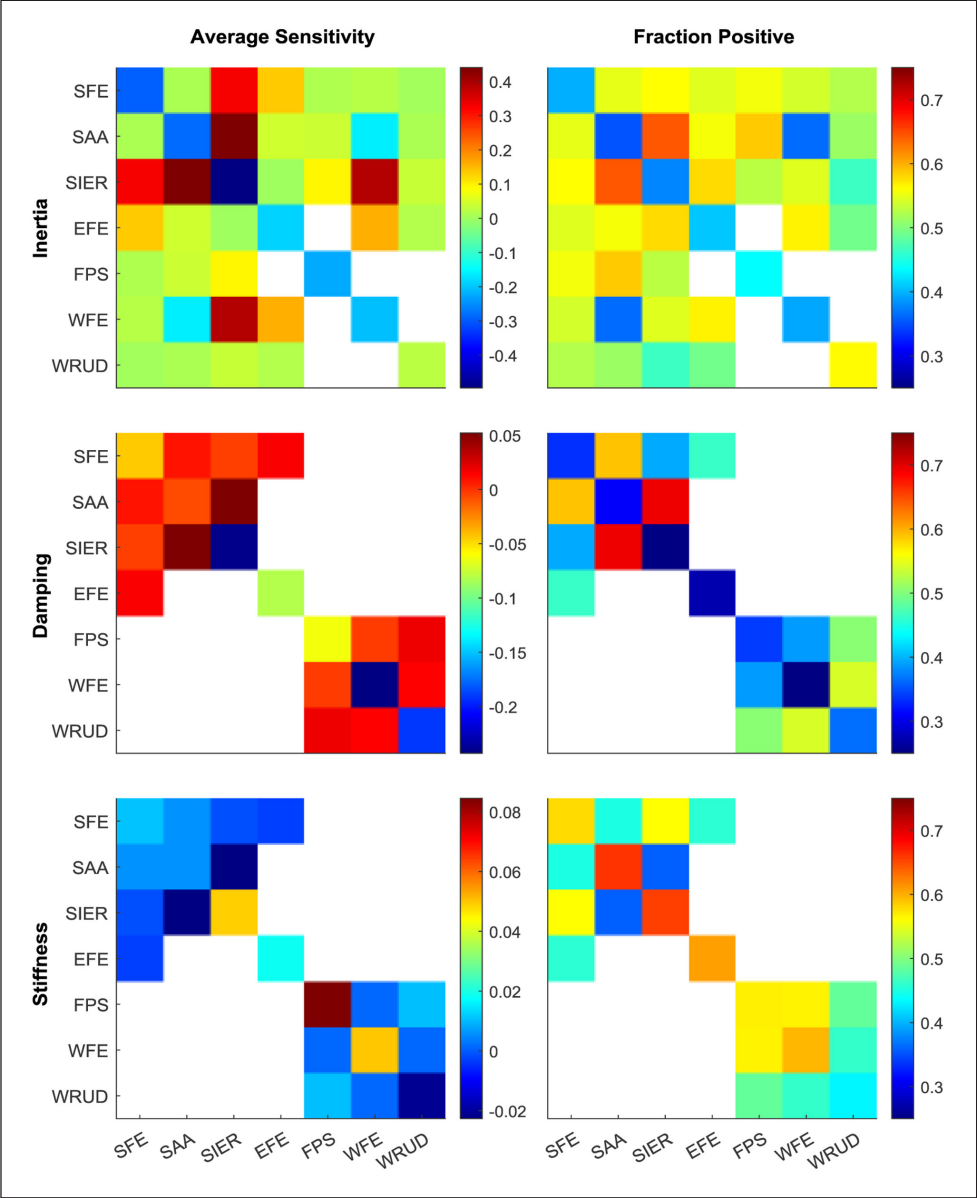
Normalized sensitivity of gains to each element of the inertia, damping, and stiffness matrices (averaged across all 350 input-output relationships and all postures). Left column: mean normalized sensitivity to each matrix element. Right column: fraction of the input-output relationships whose sensitivity is positive, indicating that an increase in the indicated element of inertia, damping, or stiffness matrices causes an increase in gain. Sensitivities associated with matrix elements assumed to be zero were left blank. Different inertia values were non-zero in different postures, so the elements of the average sensitivity and fraction positive heatmaps for inertia represent the postures in which that matrix element was non-zero.

The sensitivity analysis showed that increasing individual elements of the joint inertia, damping, or stiffness matrices increased gains for about half of the muscle-DOF-posture relationships (52%, 44%, and 51% for inertia, damping, and stiffness, respectively). Furthermore, we found that increasing any element of any of the three impedance matrices increased gains in at least 22% of muscle-DOF relationships (Figure 5). A deeper exploration revealed that although increasing damping sometimes increased gains in some DOF, it always decreased gains in the joint DOF in which it was increased (i.e. increasing elbow damping always decreased elbow tremor). In contrast, increasing joint inertia and stiffness sometimes increased and sometimes decreased gains, even in the joints in which they were increased. Gains were most sensitive to changes in inertia, then damping, then stiffness; averaging the absolute value of sensitivity over all sensitivities, gains were 4.2 times more sensitive to inertia than to damping, and 1.8 times more sensitive to damping than to stiffness. In summary, these findings confirm that increasing inertia, damping, or stiffness does not always decrease gains in all DOF, so care must be exercised in using weights or viscoelastic bracing to suppress tremor.

## 4 Discussion

### 4.1 Revised Principles of Tremor Propagation from Neural Drive to Joint Rotation

As stated above, we previously used a limited model with only 15 upper-limb muscles to postulate fundamental principles of tremor propagation from neural drive to tremulous joint rotations [38]. The purpose of this current study was to expand the model to include all 50 muscles of the upper limb (excluding intrinsic hand muscles), and to use this expanded model to simulate tremor propagation and test the validity of (and update, if necessary) the previously postulated principles of tremor propagation. Combined with robustness and sensitivity analyses, the simulations performed with the expanded model confirmed the previously postulated principles, yielded new insights, and caused us to refine aspects of some principles. Integrating these simulation results, we propose the following, updated principles.

*Principle 1: The distribution of tremor (among the joint DOF of the upper limb) depends strongly on musculoskeletal dynamics, not just on which muscle has the most tremorogenic activity*. The results of the expanded model reaffirmed that some input-output pairs have far larger gains than others (Figure 3), indicating that an individual’s neuromusculoskeletal system significantly shapes his/her tremor throughout the upper limb. As for the 15-muscle model [38], the transformation from tremorogenic neural drive to tremulous joint rotation should be viewed as a multiple-input multiple-output process that is far from dynamically transparent; the system both low-pass filters and mixes the inputs to a significant extent. Consequently, an individual’s tremor distribution cannot be predicted from sEMG recordings alone, nor is tremor in a DOF caused simply or solely by the muscles that cross that DOF.

*Principle 2: Tremor spreads (from tremorogenic activity in a given muscle to joint DOF throughout the upper limb) because of multi-articular muscles and interaction torques*. As illustrated in Figure 3, the musculoskeletal geometry of the upper limb transforms force in 50-dimensional muscle space to torque in 7-dimensional joint space, spreading force in individual muscles across multiple joint DOF in the process. The mechanical impedance of the musculoskeletal system further transforms torque into joint rotation (both in 7-dimensional joint space). Because joint inertia, damping, and stiffness couple joint DOF, rotation in a single DOF creates interaction torques in other DOF, causing secondary joint rotations and further spreading. In this manner, tremorogenic activity in a single muscle causes joint torques in multiple DOF and subsequently joint rotation in most or all DOF.

We previously attempted to quantitatively compare the amount of spreading caused by the inertia, damping, stiffness, and moment-arm matrices by comparing the effects of diagonalizing the inertia, damping, and stiffness matrices and “quasi-diagonalizing” the moment-arm matrix [38,39]. However, since the moment-arm matrix transforms between different spaces than the inertia, damping, and stiffness matrices, and without an accepted method for quantitatively defining the amount of spreading, we forebear from a quantitative comparison here. However, we note the following qualitative comparison. Since joint damping and stiffness largely reflect muscle damping and stiffness (at least away from the ends of the range of joint motion, where ligaments dominate), the spreading caused by the damping and stiffness matrices is due to multi-articular muscles. Therefore, the extent to which damping, stiffness, and moment arms spread tremor is spatially limited because muscles cross neighboring DOF. In contrast, inertia is capable of spreading tremor to DOF that are remote from each other. For example, in Posture 1, shoulder internal-external rotation and wrist flexion-extension have parallel axes of rotation, so tremor in shoulder internal-external rotation spreads to wrist flexion-extension (because of inertial coupling) even though these two DOF are far from each other and share no muscles (see Figure SM1).

*Principle 3: Tremor spreads narrowly, but not necessarily locally: although tremorogenic activity in a given muscle creates tremor in most or all DOF, most of the tremor caused by a given muscle occurs in a small number of DOF, which are not necessarily close to the muscle*. The pattern observed for 15 muscles holds true for all 50 muscles: as seen in Figure 3, the vast majority of the tremor caused by a muscle focuses in one or at most two DOF, which may or may not be crossed by that muscle. For example, even though the triceps longus muscle does not cross the wrist joint, it causes more tremor in wrist flexion-extension than any other DOF (see Figure 3). It is remarkable that this principle holds for all 50 muscles; although the musculoskeletal system causes tremorogenic force in a single muscle to spread to most or all DOF, not one of the 50 muscles creates tremor that is remotely uniform across the 7 DOF.

*Principle 4: In most postures, the largest gains occur between inputs in forearm and wrist muscles and outputs in forearm and wrist DOF*.^4^ Averaged across the postures included in this study, the largest gains (between neural drive and joint rotation) occurred from the biceps longus, pronator teres, and supinator muscles to pronation-supination; from flexor carpi radialis and ulnaris muscles to wrist flexion-extension; and from flexor carpi ulnaris to wrist radial-ulnar deviation (Figure 3). That said, the expanded model revealed large gains between many shoulder muscles and distal DOF (especially wrist flexion-extension), particularly in certain postures (e.g. postures 4–6, as seen in Figures SM4-6 in Supplemental Materials). Similarly, large gains were seen between extrinsic hand flexors and wrist flexion-extension.

Interestingly, even though the largest gains from neural drive to muscle force occurred in shoulder/elbow muscles and the largest gains from neural drive to joint torque occurred in shoulder/elbow DOF, the largest gains from neural drive to joint rotation occurred in forearm and wrist DOF (Figure 3). This whip-like effect, in which gains from neural drive or force to motion increase proximal-distally, likely reflects the proximal-distal decrease in inertia (and increase in damping and stiffness relative to inertia [70]) of open kinematic chains such as whips and the upper limb.

What is the biomechanical impact of tremorogenic muscle activation to shoulder vs elbow vs forearm vs wrist vs hand muscles? This can be read off of the heatmap of gains from neural drive to joint rotation (Figure 3) by focusing on specific sections. Averaged across the postures included in this study, shoulder muscles (DELT1 to TRIlong, plus BIClong and BICshort) have the largest gains to wrist flexion-extension, shoulder internal-external rotation, and forearm pronation-supination; elbow muscles (TRIlong to BRD) in forearm pronation-supination; forearm muscles (SUP, BIClong, BICshort, PT, PQ) in forearm pronation-supination; wrist muscles (ECRL to PL) in wrist flexion-extension and radial-ulnar deviation; and extrinsic hand muscles (FDSL to APL) in wrist flexion-extension (note that the DOF in the hand were excluded in this study). Thus, the proximal-distal trend in input-output relationships is not symmetrical: although the largest gains involving inputs to proximal muscles generally (i.e. averaged across the postures included in this study) occurred in outputs to distal DOF, the largest gains involving inputs to distal muscles did not generally occur in outputs to proximal DOF, but rather to distal DOF. These effects are summarized in Figure SM8 in Supplemental Material.

*Principle 5: Although inertial weighting or viscoelastic bracing are sometimes used to suppress tremor, increasing elements of inertia, damping, or stiffness can actually increase tremor*. Whereas inertial and viscoelastic effects were previously split into two separate principles [38,39], here we have combined the two principles into one. Using the 15-muscle model and a sensitivity analysis that emphasized the scaling of entire impedance matrices, we previously found that whereas increasing inertia or stiffness could decrease or increase tremor (depending on various factors), increasing damping always decreased tremor. However, with the 50-muscle model and a more extensive sensitivity analysis, in which we computed the sensitivity of every input-output (i.e. muscle-DOF) relationship to every impedance parameter, we found that although increasing damping always decreased tremor in the joint DOF in which it was increased, it could increase tremor in other DOF. In fact, we found that increasing any element of any of the three impedance matrices increased gains in at least some input-output relationships. In addition to these similarities, we also found differences between the three impedance matrices: on average, gains were more sensitive to inertia than to damping, and more sensitive to damping than to stiffness.

### 4.2 Limitations

In this study, we focused on the relationship from tremorogenic neural drive to tremor in joint DOF, not tremor of the hand. The amount of tremor in different joint DOF tends to differ somewhat between tremor-causing disorders, so understanding the potential of each muscle to contribute to tremor in joint DOF is clinically relevant [71]. That said, tremor of the hand is usually of more consequence to patients. The principles of tremor propagation stated in this paper refer to propagation to joint tremor and cannot be extrapolated to hand tremor. Even though joint tremor causes hand tremor, the majority of the principles stated above do not apply to hand tremor. For example, many of the principles describe the phenomenon of tremor spreading (where tremorogenic activity in a muscle spreads unequally to joint DOF throughout the upper limb), which is not relevant to hand tremor. Therefore, more research is needed to develop principles of tremor propagation specific to hand tremor.

Simulations were performed using a model that is linear and time-invariant. Although the neuromusculoskeletal system of the upper limb is generally neither linear nor time-invariant, postural tremor involves relatively small joint rotations^1^ about an equilibrium posture and does not necessarily involve significant changes in neuromusculoskeletal properties while maintaining a given posture, so it is well-suited to be approximated as a linear, time-invariant system.

Although the model consists of accepted submodels widely used in motor control research, the use of the entire model to simulate tremor propagation has not been validated. We have attempted to mitigate uncertainty by simulating only postural tremor (likely well suited to linear, time-invariant modeling) and performing a thorough robustness analysis.

The model does not include afferent feedback paths known to exist. These feedback paths likely have an effect on tremor, though the effect is unknown. That said, in preliminary investigations using a model with feedback (not shown), we have found that the effect of feedback at tremor frequencies is quite limited; in particular, feedback does not appear to have a significant effect on *patterns* of tremor propagation (i.e. which muscles produce the most tremor, or which DOF exhibit the most tremor) [72].

The DOF in the fingers were not included in the model to maintain tractability, and because model parameters (joint inertia, damping, stiffness) for individual fingers have not been sufficiently characterized. Consequently, the model simulates upper-limb tremor as if the fingers were immobile. This is a valid assumption for many activities of daily living that significantly limit finger movement, such as pinching a key or grasping a cup. Even if the fingers are free to tremble, the inertia of individual fingers is so small that it is doubtful whether finger tremor would have a significant effect on tremor in the other upper-limb DOF.

Muscle forces were calculated as a function of maximum muscle force, muscle time constants, and neural drive only. In other words, our model ignores the effects of muscle length and velocity on muscle force, assuming instead that muscles’ maximum force-generating capacity is the same in the different postures included in this study. We do not expect this to have a large impact on simulations of how musculoskeletal mechanics filter and mix tremorogenic activity on its way to becoming tremor within a given posture, where changes in muscle length are small. However, it does affect the relative gains between muscles within a given posture, and of a given muscle between different postures (particularly for proximal muscles, since the postures included in this study did not change dramatically for distal DOF). Unfortunately, the size of this effect is currently unknown.

In this study, we assessed the capacity of individual muscles to generate tremor in isolation. This does not take into account the constructive or destructive interference of joint torques caused by different muscles acting simultaneously. In reality, interference has a significant effect on the resulting tremor. However, since interference depends strongly on the relative magnitudes and phases of tremorogenic activity between muscles, which vary between subjects and even within a subject over time [58,73,74], it is difficult to make general statements about the capacity of individual muscles to contribute to the total tremor (caused by all muscles) in a given joint DOF. Similarly, it is difficult to make general statements about the effect of interventions to individual muscles on total tremor. Instead, we chose to present the gains of individual muscle-DOF relationships, which portray each muscle’s capacity for generating tremor in isolation. That said, knowing an individual subject’s model inputs (amount and phase of tremorogenic activity to each muscle), one could use this model to estimate the contribution of each muscle to (and predict the effect of interventions on) total tremor.

### 4.3 Conclusion

The purpose of this study was to expand a previous tremor propagation model with only 15 upper-limb muscles to include all 50 upper-limb muscles (excluding intrinsic hand muscles), and to use this expanded model to simulate tremor propagation and test the validity of (and update, if necessary) the previously postulated principles of tremor [38,39]. Simulations with the expanded model confirmed previously postulated principles, revealed several new insights, and caused us to refine aspects of several principles, including: 1) most of the muscles with the greatest capacity for causing tremor are among the 15 major superficial muscles included in the original model; 2) some gains depended strongly on the posture of the limb; 3) averaged across the postures included in this study, the largest gains belonged to input-output pairs involving biceps/forearm/wrist muscles and forearm/wrist DOF; 4) some shoulder and extrinsic hand muscles were also capable of causing significant tremor, especially in certain postures; and 5) although inertial weighting or viscoelastic bracing are sometimes used to suppress tremor, increasing joint inertia, damping, or stiffness matrices can also increase gains, so care must be taken.

Importantly, these observations suggest that a) the most important tremorogenic activity may be captured by recording from the 15 major superficial muscles of the upper limb alone, b) in the absence of additional information (such as tremorogenic neural drive to muscles), peripheral tremor suppression efforts should start by targeting biceps/forearm/wrist muscles or forearm/wrist DOF, and c) gains involving a specific joint are decreased by increasing damping in that joint, but determining the net impact of this damping on tremor at the hand requires further investigation.

Tremor depends not only on musculoskeletal dynamics, but also on action, intention, and psychological factors in complex ways that are not well understood. We hope that understanding the effect of musculoskeletal dynamics on tremor can remove an unknown and make it easier to disentangle the effects of other factors, eventually leading to an understanding of how various factors combine to create the complex nature of tremor observed in every-day life.

## Additional File

The additional file for this article can be found as follows:

10.5334/tohm.949.s1 Supplementary material. Tables SM1 to SM11 and Figures SM1 to SM9.

## Footnotes

In a re-analysis (not shown) of the tremor data described in [76], we found that even subjects with severe tremor (according to their TETRAS scores) had mean joint tremor amplitudes of only 0.5–2.5 degrees (depending on the DOF), even when tremor amplitude was generously defined as the difference between the minimum and maximum joint angles recorded over a 30s trial. This amplitude is far smaller than thresholds commonly used in engineering and science to justify small-angle approximations of non-linear systems as linear [77].

These basic viscoelastic effects captured in the damping and stiffness matrices refer to the changes in muscle force caused by small perturbations to the length or rate of change of length of a muscle after it has been activated in a given posture. They do *not* refer to what are commonly known as the force-length and force-velocity relationships of muscle, which capture the change in force-generating capacity caused by activating a muscle at a different length or rate of change of length (see Limitations).

Alternatively, it may be more intuitive to interpret the pattern of gains as the relative muscle forces, joint torques, and joint rotations that would result if all muscles received the same amount of tremorogenic input (e.g. 10% of MVC).

Alternatively, the following interpretation may be more intuitive: Assuming that all muscles receive the same amount of tremorogenic activity (e.g. 10% of MVC), tremor is greatest in the wrist and forearm, and most of the tremor in the wrist and forearm is caused by muscles that cross the wrist and forearm.
