# Supplementary material for: Potential of Individual Upper-Limb Muscles to Contribute to Postural Tremor: Simulations From Neural Drive to Joint Rotation
Source: Tremor Other Hyperkinet Mov (N Y). 2025 Feb 25;15:7. doi: 10.5334/tohm.949 (PMC11869822; doi:10.5334/tohm.949)
Supplement: Supplementary material. — Tables SM1 to SM11 and Figures SM1 to SM9. [file tohm-15-1-949-s1.pdf]

Supplemental Material

Tables

Table SM1. Damping matrix used for Postures 1-6. Diagonal elements indicate damping within a DOF, whereas off diagonal terms couple damping between DOF. The abbreviations represent shoulder flexion-extension (SFE), shoulder abduction-adduction (SAA), shoulder internal-external rotation (SIER), elbow flexion-extension (EFE), forearm pronation-supination (FPS), wrist flexion-extension (WFE), and wrist radial-ulnar deviation (WRUD). Posture 7 used this same matrix, with the EFE-EFE element of the matrix increased by a factor of 100. Values from [1].

Damping (D) [N*m*s/rad]							
	SFE	SAA	SIER	EFE	FPS	WFE	WRUD
SFE	0.756	0.184	0.020	0.187	0	0	0
SAA	0.184	0.383	0.267	0	0	0	0
SIER	0.020	0.267	0.524	0	0	0	0
EFE	0.187	0	0	0.607	0	0	0
FPS	0	0	0	0	0.021	0.000	0.008
WFE	0	0	0	0	0.000	0.028	-0.003
WRUD	0	0	0	0	0.008	-0.003	0.082

Table SM2. Stiffness matrix used for Postures 1-6. Diagonal elements indicate stiffness within a DOF, whereas off diagonal terms couple stiffness between DOF. Posture 7 used this same matrix, with the EFE-EFE element of the matrix increased by a factor of 10,000. Values from [1].

Stiffness (K) [N*m/rad]							
	SFE	SAA	SIER	EFE	FPS	WFE	WRUD
SFE	10.800	2.626	0.279	2.670	0	0	0
SAA	2.626	5.469	3.821	0	0	0	0
SIER	0.279	3.821	7.486	0	0	0	0
EFE	2.670	0	0	8.670	0	0	0
FPS	0	0	0	0	0.756	0.018	0.291
WFE	0	0	0	0	0.018	0.992	-0.099
WRUD	0	0	0	0	0.291	-0.099	2.920

Table SM3. Masses and heights used to scale subject size for the robustness analysis. Based on the United States 2015-2018 anthropometric reference data [2]. Outside of the robustness analysis, the average of 50th percentile male and female parameters were used.

	Mass [kg]		Height [cm]	
Percentile	Male	Female	Male	Female
10	66.6	53.9	165.8	152.5
50	87.4	73.1	175.4	161.3
90	119.4	105.3	184.7	170.2

Table SM4. Normalized segment length, mass, center of mass (CM), and radii of gyration for the upper arm, forearm, and hand. The segment lengths and masses are expressed as a percentage of subject parameters (height and mass respectively), while all other parameters are expressed as a percentage of segment length. The center of mass was measured from the more proximal of the joints bounding each link. The radius of gyration is measured from the center of mass, with the coordinate frame of that link shown in [1]. Outside of the robustness analysis, only the values in the average row were used. Values derived from [3].

	Upper Arm			Forearm			Hand		
	Male	Female	Average	Male	Female	Average	Male	Female	Average
Length (%)	0.162	0.159	0.160	0.155	0.152	0.153	0.050	0.045	0.047
Mass (%)	0.0271	0.0252	0.0262	0.0162	0.0150	0.0156	0.0061	0.0057	0.0059
CM (%)	0.577	0.577	0.577	0.457	0.457	0.457	0.790	0.790	0.790
r - x (%)	0.285	0.278	0.282	0.276	0.261	0.269	0.401	0.335	0.368
r - y (%)	0.158	0.148	0.153	0.121	0.094	0.108	0.513	0.454	0.484
r - z (%)	0.269	0.26	0.265	0.265	0.257	0.261	0.628	0.531	0.580

Table SM5. Transpose of moment arm matrix in Posture 1. Moment arms are given in millimeters. Negative moment arms indicate that neural drive to a given muscle creates torque in the negative direction. Positive directions are shoulder flexion, adduction, and internal rotation; elbow flexion; forearm pronation; and wrist flexion and ulnar deviation. In Posture 1, the shoulder is in gimbal lock, so all moment arms with respect to SFE were set to zero (see [4] for details).

Muscle	SFE	SAA	SIER	EFE	FPS	WFE	WRUD
DELT1	0	12.1	4.4	0	0	0	0
DELT2	0	-23.2	0.7	0	0	0	0
DELT3	0	-16.6	-8.0	0	0	0	0
SUPSP	0	-18.2	-4.1	0	0	0	0
INFSP	0	-3.3	-18.9	0	0	0	0
SUBSC	0	-9.8	13.3	0	0	0	0
TMIN	0	5.8	-15.2	0	0	0	0
TMAJ	0	5.7	7.8	0	0	0	0
PECM1	0	28.8	10.3	0	0	0	0
PECM2	0	51.3	8.0	0	0	0	0
PECM3	0	51.7	-1.4	0	0	0	0
LAT1	0	-4.6	9.3	0	0	0	0
LAT2	0	-7.6	8.3	0	0	0	0
LAT3	0	4.2	5.8	0	0	0	0
CORB	0	30.5	1.5	0	0	0	0
TRIlong	0	6.9	-4.9	-16.5	0	0	0
TRIlat	0	0	0	-16.5	0	0	0
TRImed	0	0	0	-16.5	0	0	0
ANC	0	0	0	-9.1	0	0	0
SUP	0	0	0	0	-10.9	0	0
BIClong	0	-4.1	5.0	47.0	-13.4	0	0
BICshort	0	33.6	4.6	47.0	-13.4	0	0
BRA	0	0	0	23.5	0	0	0
BRD	0	0	0	69.7	5.3	0	0
ECRL	0	0	0	24.5	2.0	-9.7	-21.0
ECRB	0	0	0	-1.9	-1.0	-13.1	-12.4
ECU	0	0	0	-2.3	-0.7	-6.1	24.7
FCR	0	0	0	11.0	2.0	14.7	-7.9
FCU	0	0	0	11.6	1.2	14.9	20.8
PL	0	0	0	13.6	2.0	22.5	-3.9
PT	0	0	0	12.8	10.5	0	0
PQ	0	0	0	0	7.7	0	0
FDSL	0	0	0	12.8	1.0	14.3	6.6
FDSR	0	0	0	10.4	1.6	14.0	5.0
FDSM	0	0	0	0	3.0	14.0	-1.0
FDSI	0	0	0	0	3.3	13.7	-2.9
FDPL	0	0	0	0	2.0	11.2	4.9
FDPR	0	0	0	0	2.1	11.5	3.2
FDPM	0	0	0	0	2.6	11.4	0.9
FDPI	0	0	0	0	2.7	11.5	-3.3
EDCL	0	0	0	0.7	1.8	-11.7	10.1
EDCR	0	0	0	0.1	1.7	-15.7	7.0
EDCM	0	0	0	-0.3	-0.4	-23.8	14.0
EDCI	0	0	0	0.3	1.7	-15.7	-5.2
EDM	0	0	0	-1.3	-0.6	-10.3	12.3
EIP	0	0	0	0	-0.3	-14.1	-4.4
EPL	0	0	0	0	-1.9	-6.6	-8.4
EPB	0	0	0	0	0	3.2	-21.2
FPL	0	0	0	0	0	10.2	-7.0
APL	0	0	0	0	0	6.7	-20.3

Table SM6. Transpose of moment arm matrix in Posture 2. See the caption of **Table SM5.** Transpose of moment arm matrix in Posture 1. Moment arms are given in millimeters. Negative moment arms indicate that neural drive to a given muscle creates torque in the negative direction. Positive directions are shoulder flexion, adduction, and internal rotation; elbow flexion; forearm pronation; and wrist flexion and ulnar deviation. In Posture 1, the shoulder is in gimbal lock, so all moment arms with respect to SFE were set to zero (see [4] for details).Table SM5 for full description.

Muscle	SFE	SAA	SIER	EFE	FPS	WFE	WRUD
DELT1	46.9	4.4	3.7	0	0	0	0
DELT2	19.6	-9.9	-8.3	0	0	0	0
DELT3	-17.6	-2.3	-7.2	0	0	0	0
SUPSP	3.4	-10.9	1.1	0	0	0	0
INFSP	7.0	-2.4	-15.1	0	0	0	0
SUBSC	-1.3	-9.0	2.2	0	0	0	0
TMIN	-2.6	4.3	-10.0	0	0	0	0
TMAJ	-30.7	5.1	14.6	0	0	0	0
PECM1	8.7	12.0	19.9	0	0	0	0
PECM2	-19.2	7.7	13.6	0	0	0	0
PECM3	-31.8	4.7	10.7	0	0	0	0
LAT1	-26.8	0.7	11.0	0	0	0	0
LAT2	-29.5	-0.7	9.0	0	0	0	0
LAT3	-39.9	-2.7	7.4	0	0	0	0
CORB	-8.1	11.5	12.9	0	0	0	0
TRIlong	-20.1	9.0	1.4	-16.3	0	0	0
TRIlat	0	0	0	-16.3	0	0	0
TRImed	0	0	0	-16.3	0	0	0
ANC	0	0	0	-10.7	0	0	0
SUP	0	0	0	0	-7.8	0	0
BIClong	8.3	-5.4	7.9	35.1	-3.9	0	0
BICshort	-16.4	11.4	16.9	35.1	-3.9	0	0
BRA	0	0	0	23.9	0	0	0
BRD	0	0	0	71.3	17.9	0	0
ECRL	0	0	0	25.7	5.6	-9.7	-21.0
ECRB	0	0	0	-4.2	-1.0	-13.1	-12.4
ECU	0	0	0	1.1	1.4	-6.1	24.7
FCR	0	0	0	5.9	5.2	14.7	-7.9
FCU	0	0	0	9.9	2.8	14.9	20.8
PL	0	0	0	9.2	6.1	22.5	-3.9
PT	0	0	0	10.8	9.0	0	0
PQ	0.0	0.0	0.0	0.0	4.5	0	0
FDSL	0	0	0	9.4	4.4	14.3	6.6
FDSR	0	0	0	7.3	4.5	14.0	5.0
FDSM	0	0	0	0	3.4	14.0	-1.0
FDSI	0	0	0	0	3.4	13.7	-2.9
FDPL	0	0	0	0	2.2	11.2	4.9
FDPR	0	0	0	0	2.4	11.5	3.2
FDPM	0	0	0	0	2.7	11.4	0.9
FDPI	0	0	0	0	2.5	11.5	-3.3
EDCL	0	0	0	4.4	1.3	-11.7	10.1
EDCR	0	0	0	4.2	1.5	-15.7	7.0
EDCM	0	0	0	3.0	0.4	-23.4	10.8
EDCI	0	0	0	3.5	1.4	-15.7	-5.2
EDM	0	0	0	2.1	-0.1	-10.3	12.3
EIP	0	0	0	0	-0.2	-14.1	-4.4
EPL	0	0	0	0	-1.2	-6.6	-8.4
EPB	0	0	0	0	0	3.2	-21.2
FPL	0	0	0	0	0	10.2	-7.0
APL	0	0	0	0	0	6.7	-20.3

Table SM7. Transpose of moment arm matrix in Posture 3. See the caption of Table SM5 for full description.

Muscle	SFE	SAA	SIER	EFE	FPS	WFE	WRUD
DELT1	19.2	-14.6	9.9	0	0	0	0
DELT2	17.3	-17.5	3.0	0	0	0	0
DELT3	-9.7	6.3	-14.7	0	0	0	0
SUPSP	9.1	-8.5	9.6	0	0	0	0
INFSP	5.4	-5.1	-12.1	0	0	0	0
SUBSC	6.3	-7.0	0.6	0	0	0	0
TMIN	-5.7	5.0	-7.2	0	0	0	0
TMAJ	-19.5	17.7	1.3	0	0	0	0
PECM1	0.8	2.9	6.7	0	0	0	0
PECM2	-9.8	13.4	3.0	0	0	0	0
PECM3	-5.7	9.5	-1.0	0	0	0	0
LAT1	-13.9	11.9	3.2	0	0	0	0
LAT2	-14.1	11.7	0.8	0	0	0	0
LAT3	-17.6	15.5	0.2	0	0	0	0
CORB	-7.6	10.9	7.7	0	0	0	0
TRIlong	-15.7	14.4	-13.8	-16.5	0	0	0
TRIlat	0	0	0	-16.5	0	0	0
TRImed	0	0	0	-16.5	0	0	0
ANC	0	0	0	-9.1	0	0	0
SUP	0	0	0	0	-10.9	0	0
BIClong	3.9	-2.3	15.2	47.0	-14.0	0	0
BICshort	-8.7	12.3	5.5	47.0	-14.0	0	0
BRA	0	0	0	23.5	0	0	0
BRD	0	0	0	69.7	5.3	0	0
ECRL	0	0	0	24.5	2.0	-9.7	-21.0
ECRB	0	0	0	-1.9	-1.0	-13.1	-12.4
ECU	0	0	0	-2.3	-0.7	-6.1	24.7
FCR	0	0	0	11.0	2.0	14.7	-7.9
FCU	0	0	0	11.6	1.2	14.9	20.8
PL	0	0	0	13.6	2.0	22.5	-3.9
PT	0	0	0	12.8	10.5	0	0
PQ	0	0	0	0	7.7	0	0
FDSL	0	0	0	12.8	1.0	14.3	6.6
FDSR	0	0	0	10.4	1.6	14.0	5.0
FDSM	0	0	0	0	3.0	14.0	-1.0
FDSI	0	0	0	0	3.3	13.7	-2.9
FDPL	0	0	0	0	2.0	11.2	4.9
FDPR	0	0	0	0	2.1	11.5	3.2
FDPM	0	0	0	0	2.6	11.4	0.9
FDPI	0	0	0	0	2.7	11.5	-3.3
EDCL	0	0	0	0.7	1.8	-11.7	10.1
EDCR	0	0	0	0.1	1.7	-15.7	7.0
EDCM	0	0	0	-0.3	-0.4	-23.8	14.0
EDCI	0	0	0	0.3	1.7	-15.7	-5.2
EDM	0	0	0	-1.3	-0.6	-10.3	12.3
EIP	0	0	0	0	-0.3	-14.1	-4.4
EPL	0	0	0	0	-1.9	-6.6	-8.4
EPB	0	0	0	0	0	3.2	-21.2
FPL	0	0	0	0	0	10.2	-7.0
APL	0	0	0	0	0	6.7	-20.3

Table SM8. Transpose of moment arm matrix in Posture 4. See the caption of Table SM5 for full description.

Muscle	SFE	SAA	SIER	EFE	FPS	WFE	WRUD
DELT1	41.8	4.4	22.7	0	0	0	0
DELT2	19.1	-13.2	-4.0	0	0	0	0
DELT3	-16.3	-3.9	-15.3	0	0	0	0
SUPSP	4.9	-12.4	-2.2	0	0	0	0
INFSP	6.5	-3.0	-14.2	0	0	0	0
SUBSC	-1.5	-8.1	6.0	0	0	0	0
TMIN	-2.0	4.2	-13.0	0	0	0	0
TMAJ	-29.0	7.2	5.2	0	0	0	0
PECM1	6.4	12.2	25.8	0	0	0	0
PECM2	-20.1	15.7	17.7	0	0	0	0
PECM3	-34.6	16.1	11.7	0	0	0	0
LAT1	-25.0	3.1	3.1	0	0	0	0
LAT2	-28.1	1.9	0.6	0	0	0	0
LAT3	-39.0	3.1	-1.9	0	0	0	0
CORB	-9.7	17.5	14.7	0	0	0	0
TRIlong	-21.0	8.6	-5.7	-18.2	0	0	0
TRIlat	0	0	0	-18.2	0	0	0
TRImed	0	0	0	-18.2	0	0	0
ANC	0	0	0	-10.2	0	0	0
SUP	0	0	0	0	-8.7	0	0
BIClong	10.4	-5.3	7.9	43.7	-10.8	0	0
BICshort	-16.5	17.2	16.5	43.7	-10.8	0	0
BRA	0	0	0	20.7	0	0	0
BRD	0	0	0	58.6	10.0	0	0
ECRL	0	0	0	19.8	3.2	-9.7	-21.0
ECRB	0	0	0	-0.3	-0.5	-13.1	-12.4
ECU	0	0	0	-3.5	0.8	-6.1	24.7
FCR	0	0	0	11.2	4.1	14.7	-7.9
FCU	0	0	0	10.7	2.3	14.9	20.8
PL	0	0	0	13.2	4.7	22.5	-3.9
PT	0	0	0	11.9	9.8	0	0
PQ	0	0	0	0	5.8	0	0
FDSL	0	0	0	12.3	3.2	14.3	6.6
FDSR	0	0	0	10.1	3.6	14.0	5.0
FDSM	0	0	0	0	3.5	14.0	-1.0
FDSI	0	0	0	0	3.6	13.7	-2.9
FDPL	0	0	0	0	2.3	11.2	4.9
FDPR	0	0	0	0	2.5	11.5	3.2
FDPM	0	0	0	0	2.8	11.4	0.9
FDPI	0	0	0	0	2.7	11.5	-3.3
EDCL	0	0	0	-1.0	1.1	-11.7	10.1
EDCR	0	0	0	-1.9	1.1	-15.7	7.0
EDCM	0	0	0	-1.9	-0.3	-23.6	12.0
EDCI	0	0	0	-1.2	1.2	-15.7	-5.2
EDM	0	0	0	-2.5	-0.3	-10.3	12.3
EIP	0	0	0	0	-0.3	-14.1	-4.4
EPL	0	0	0	0	-1.5	-6.6	-8.4
EPB	0	0	0	0	0	3.2	-21.2
FPL	0	0	0	0	0	10.2	-7.0
APL	0	0	0	0	0	6.7	-20.3

Table SM9. Transpose of moment arm matrix in Posture 5. See the caption of Table SM5 for full description.

Muscle	SFE	SAA	SIER	EFE	FPS	WFE	WRUD
DELT1	66.2	25.3	72.2	0	0	0	0
DELT2	38.8	-20.1	31.8	0	0	0	0
DELT3	-34.0	-26.1	-45.3	0	0	0	0
SUPSP	20.6	-14.1	12.8	0	0	0	0
INFSP	16.4	3.4	-2.6	0	0	0	0
SUBSC	1.9	-8.2	15.9	0	0	0	0
TMIN	-2.7	8.2	-18.2	0	0	0	0
TMAJ	-53.0	-3.6	-42.2	0	0	0	0
PECM1	22.7	11.9	35.2	0	0	0	0
PECM2	-22.6	23.4	-2.2	0	0	0	0
PECM3	-45.5	29.0	-21.7	0	0	0	0
LAT1	-48.3	-5.4	-36.6	0	0	0	0
LAT2	-52.9	-6.8	-41.4	0	0	0	0
LAT3	-68.7	1.1	-53.9	0	0	0	0
CORB	-24.0	37.0	-11.3	0	0	0	0
TRIlong	-43.5	-8.8	-44.4	-24.1	0	0	0
TRIlat	0	0	0	-24.1	0	0	0
TRImed	0	0	0	-24.1	0	0	0
ANC	0	0	0	-11.4	0	0	0
SUP	0	0	0	0	-7.8	0	0
BIClong	27.2	15.4	20.9	27.2	-7.4	0	0
BICshort	-25.4	28.0	-11.5	27.2	-7.4	0	0
BRA	0	0	0	11.1	0	0	0
BRD	0	0	0	22.9	3.8	0	0
ECRL	0	0	0	7.6	1.0	-9.7	-21.0
ECRB	0	0	0	3.5	0.4	-13.1	-12.4
ECU	0	0	0	-5.6	1.4	-6.1	24.7
FCR	0	0	0	8.9	4.7	14.7	-7.9
FCU	0	0	0	6.7	2.8	14.9	20.8
PL	0	0	0	8.4	5.5	22.5	-3.9
PT	0	0	0	8.1	9.0	0	0
PQ	0.0	0.0	0.0	0.0	4.5	0	0
FDSL	0	0	0	8.1	4.0	14.3	6.6
FDSR	0	0	0	8.0	4.3	14.0	5.0
FDSM	0	0	0	0	3.4	14.0	-1.0
FDSI	0	0	0	0	3.4	13.7	-2.9
FDPL	0	0	0	0	2.2	11.2	4.9
FDPR	0	0	0	0	2.4	11.5	3.2
FDPM	0	0	0	0	2.7	11.4	0.9
FDPI	0	0	0	0	2.5	11.5	-3.3
EDCL	0	0	0	-5.2	0.6	-11.7	10.1
EDCR	0	0	0	-6.3	0.5	-15.7	7.0
EDCM	0	0	0	-5.2	-0.5	-23.4	10.8
EDCI	0	0	0	-4.7	0.7	-15.7	-5.2
EDM	0	0	0	-5.2	-0.1	-10.3	12.3
EIP	0	0	0	0	-0.2	-14.1	-4.4
EPL	0	0	0	0	-1.2	-6.6	-8.4
EPB	0	0	0	0	0	3.2	-21.2
FPL	0	0	0	0	0	10.2	-7.0
APL	0	0	0	0	0	6.7	-20.3

Table SM10. Transpose of moment arm matrix in Posture 6. See the caption of Table SM5 for full description.

Muscle	SFE	SAA	SIER	EFE	FPS	WFE	WRUD
DELT1	31.7	43.5	27.1	0	0	0	0
DELT2	38.9	-10.6	28.8	0	0	0	0
DELT3	-4.6	-26.3	-9.8	0	0	0	0
SUPSP	23.7	-4.6	17.7	0	0	0	0
INFSP	14.7	5.9	-2.0	0	0	0	0
SUBSC	2.5	-7.4	15.5	0	0	0	0
TMIN	-2.3	7.8	-17.8	0	0	0	0
TMAJ	-34.8	-12.8	-18.7	0	0	0	0
PECM1	8.0	17.9	16.2	0	0	0	0
PECM2	-33.4	13.6	-12.8	0	0	0	0
PECM3	-48.6	12.7	-23.6	0	0	0	0
LAT1	-29.8	-13.1	-14.9	0	0	0	0
LAT2	-36.2	-17.1	-19.4	0	0	0	0
LAT3	-51.5	-15.6	-29.5	0	0	0	0
CORB	-40.2	30.3	-28.9	0	0	0	0
TRIlong	-20.7	-12.5	-20.0	-16.4	0	0	0
TRIlat	0	0	0	-16.4	0	0	0
TRImed	0	0	0	-16.4	0	0	0
ANC	0	0	0	-10.3	0	0	0
SUP	0	0	0	0	-10.1	0	0
BIClong	3.3	26.5	-4.5	44.8	-10.3	0	0
BICshort	-35.9	22.7	-23.8	44.8	-10.3	0	0
BRA	0	0	0	24.6	0	0	0
BRD	0	0	0	74.2	11.3	0	0
ECRL	0	0	0	26.2	3.7	-9.7	-21.0
ECRB	0	0	0	-4.3	-1.2	-13.1	-12.4
ECU	0	0	0	0.7	-0.2	-6.1	24.7
FCR	0	0	0	7.1	3.3	14.7	-7.9
FCU	0	0	0	10.7	1.9	14.9	20.8
PL	0	0	0	10.5	3.6	22.5	-3.9
PT	0	0	0	11.5	10.4	0	0
PQ	0	0	0	0	7.1	0	0
FDSL	0	0	0	10.5	2.2	14.3	6.6
FDSR	0	0	0	8.2	2.7	14.0	5.0
FDSM	0	0	0	0	3.3	14.0	-1.0
FDSI	0	0	0	0	3.5	13.7	-2.9
FDPL	0	0	0	0	2.1	11.2	4.9
FDPR	0	0	0	0	2.3	11.5	3.2
FDPM	0	0	0	0	2.7	11.4	0.9
FDPI	0	0	0	0	2.8	11.5	-3.3
EDCL	0	0	0	4.1	1.7	-11.7	10.1
EDCR	0	0	0	3.9	1.8	-15.7	7.0
EDCM	0	0	0	2.9	-0.1	-23.8	13.3
EDCI	0	0	0	3.3	1.7	-15.7	-5.2
EDM	0	0	0	1.7	-0.5	-10.3	12.3
EIP	0	0	0	0	-0.3	-14.1	-4.4
EPL	0	0	0	0	-1.8	-6.6	-8.4
EPB	0	0	0	0	0	3.2	-21.2
FPL	0	0	0	0	0	10.2	-7.0
APL	0	0	0	0	0	6.7	-20.3

Table SM11. Transpose of moment arm matrix in Posture 7. See the caption of Table SM5 for full description.

Muscle	SFE	SAA	SIER	EFE	FPS	WFE	WRUD
DELT1	37.0	24.8	-3.6	0	0	0	0
DELT2	22.2	-16.6	5.6	0	0	0	0
DELT3	-5.8	-11.9	-4.1	0	0	0	0
SUPSP	7.2	-25.2	17.6	0	0	0	0
INFSP	17.7	-13.6	-9.1	0	0	0	0
SUBSC	6.0	-12.3	6.0	0	0	0	0
TMIN	2.2	3.9	-10.8	0	0	0	0
TMAJ	-31.1	1.4	6.1	0	0	0	0
PECM1	-9.0	4.1	8.1	0	0	0	0
PECM2	-37.1	-8.6	11.0	0	0	0	0
PECM3	-44.6	-9.5	13.0	0	0	0	0
LAT1	-26.1	-0.6	6.3	0	0	0	0
LAT2	-29.5	-5.2	6.2	0	0	0	0
LAT3	-39.9	-9.4	8.7	0	0	0	0
CORB	-31.2	15.7	2.0	0	0	0	0
TRIlong	-16.8	2.2	-2.7	-25.1	0	0	0
TRIlat	0	0	0	-25.1	0	0	0
TRImed	0	0	0	-25.1	0	0	0
ANC	0	0	0	-7.5	0	0	0
SUP	0	0	0	0	-10.9	0	0
BIClong	5.4	13.5	5.4	9.6	-5.1	0	0
BICshort	-32.2	11.7	4.6	9.6	-5.1	0	0
BRA	0	0	0	9.3	0	0	0
BRD	0	0	0	11.3	-0.1	0	0
ECRL	0	0	0	5.0	-1.3	-9.7	-21.0
ECRB	0	0	0	4.8	-0.6	-13.1	-12.4
ECU	0	0	0	-5.4	-0.7	-6.1	24.7
FCR	0	0	0	6.0	2.6	14.7	-7.9
FCU	0	0	0	5.3	1.8	14.9	20.8
PL	0	0	0	1.7	3.6	22.5	-3.9
PT	0	0	0	4.2	10.5	0	0
PQ	0	0	0	0	7.7	0	0
FDSL	0	0	0	3.0	2.1	14.3	6.6
FDSR	0	0	0	2.4	2.6	14.0	5.0
FDSM	0	0	0	0	3.0	14.0	-1.0
FDSI	0	0	0	0	3.3	13.7	-2.9
FDPL	0	0	0	0	2.0	11.2	4.9
FDPR	0	0	0	0	2.1	11.5	3.2
FDPM	0	0	0	0	2.6	11.4	0.9
FDPI	0	0	0	0	2.7	11.5	-3.3
EDCL	0	0	0	-6.6	1.4	-11.7	10.1
EDCR	0	0	0	-7.5	1.2	-15.7	7.0
EDCM	0	0	0	-6.0	-0.8	-23.8	14.0
EDCI	0	0	0	-5.7	1.4	-15.7	-5.2
EDM	0	0	0	-5.6	-0.6	-10.3	12.3
EIP	0	0	0	0	-0.3	-14.1	-4.4
EPL	0	0	0	0	-1.9	-6.6	-8.4
EPB	0	0	0	0	0	3.2	-21.2
FPL	0	0	0	0	0	10.2	-7.0
APL	0	0	0	0	0	6.7	-20.3

Figures

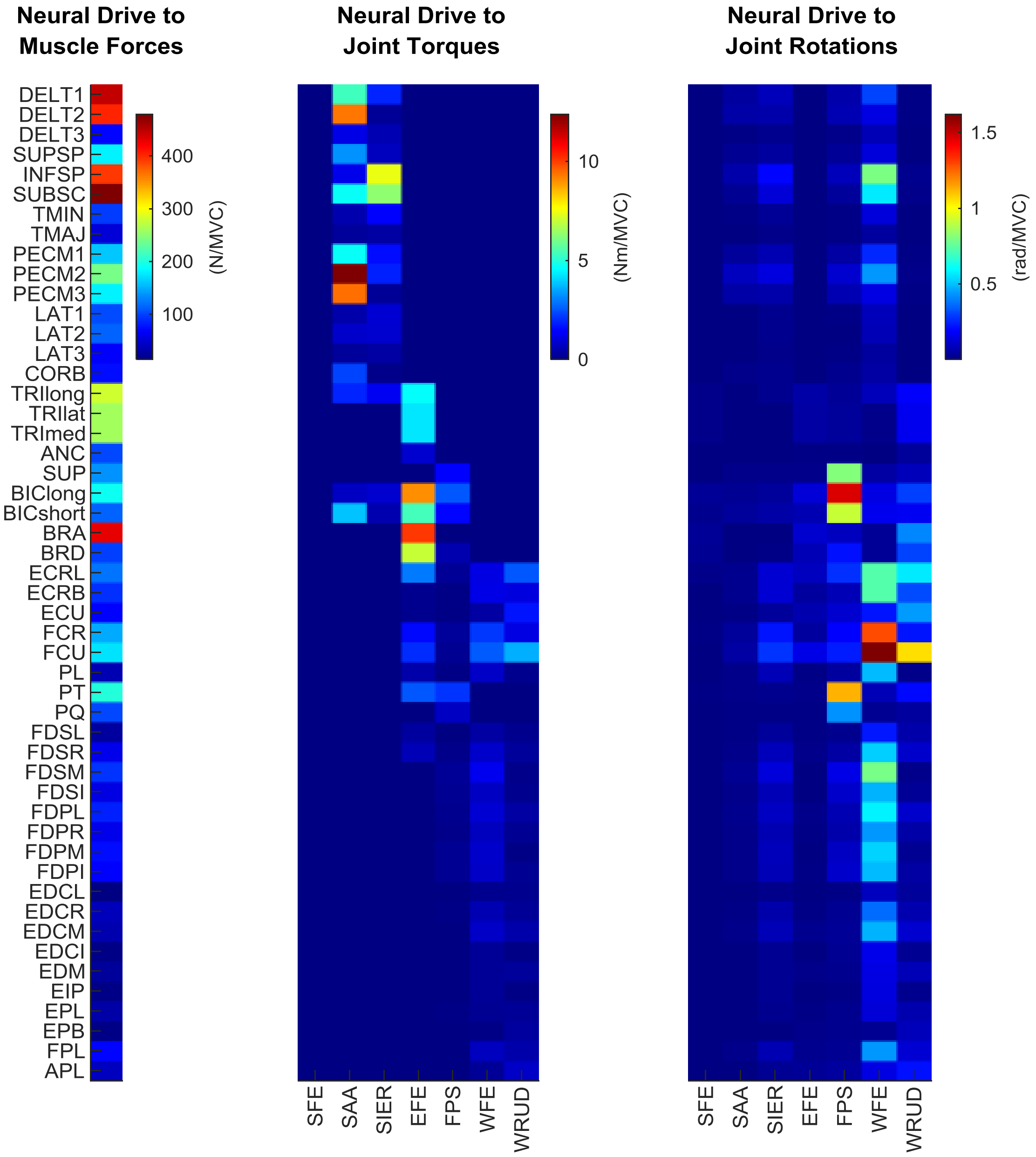

Figure SM1. Gains from neural drive to muscle force, joint torque, and joint displacement for 6 Hz tremor in Posture 1. Alternatively, the heatmaps can be interpreted as muscle force, joint torque, and joint displacement caused by the same magnitude of tremorogenic neural drive to each muscle (relative to each muscle's maximum voluntary contraction). Muscles are ordered proximal (top) to distal (bottom; for muscle names, see Table 1 in the body of the article). Similarly, joint DOF are listed proximal (left) to distal (right): shoulder flexion-extension (SFE), abduction-adduction (SAA), and internal-external rotation (SIER); elbow flexion-extension (EFE) and forearm pronation-supination (FPS); and wrist flexion-extension (WFE) and radial-ulnar deviation (WRUD), respectively. In Posture 1 only, the shoulder is in gimbal lock, so all moment arms with respect to SFE were set to zero, resulting in zero joint torque in SFE (see [4] for details).

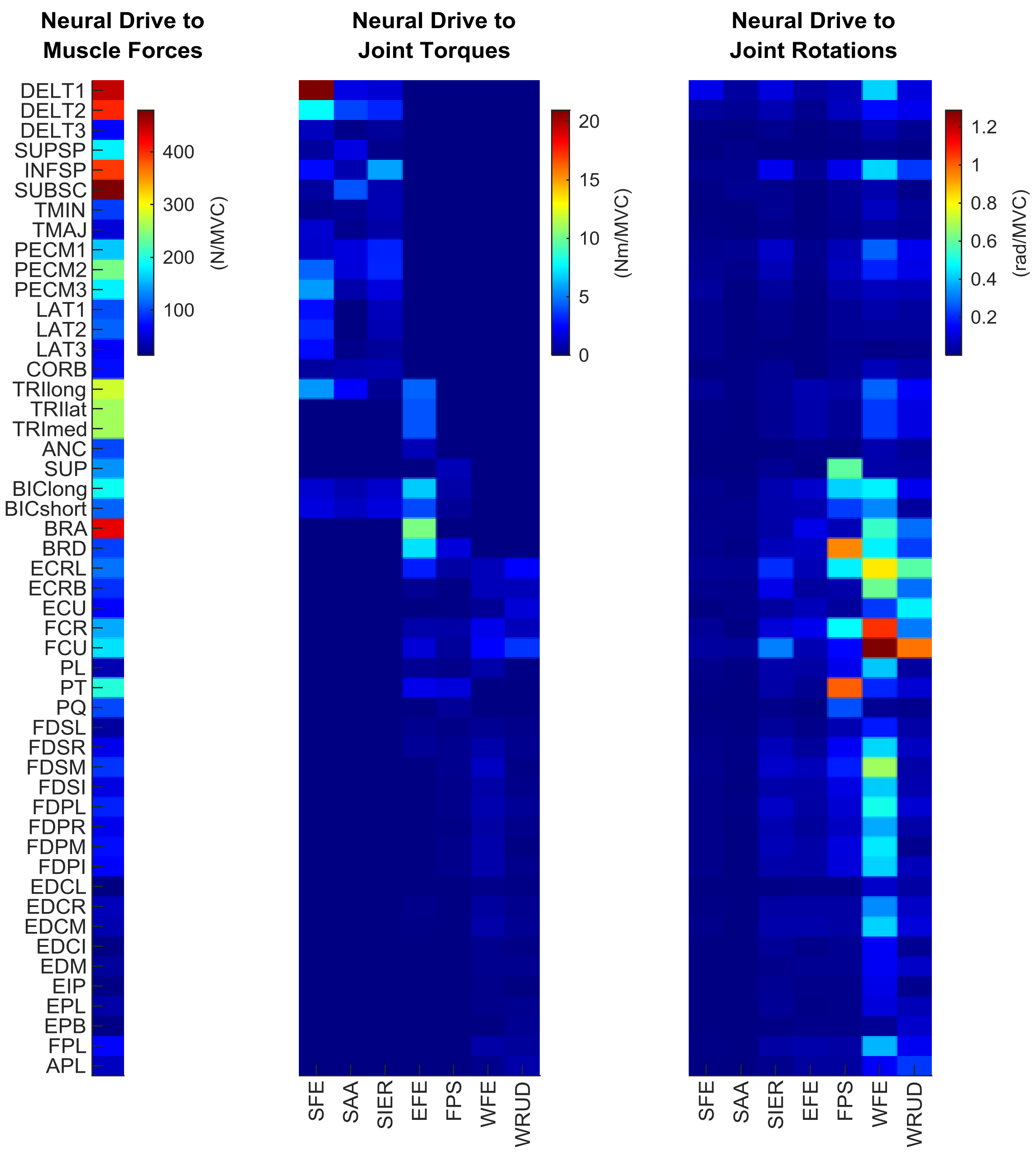

Figure SM2. Gains from neural drive to muscle force, joint torque, and joint displacement for 6 Hz tremor in Posture 2. See the caption to ***Error! Reference source not found.***1 for more details.

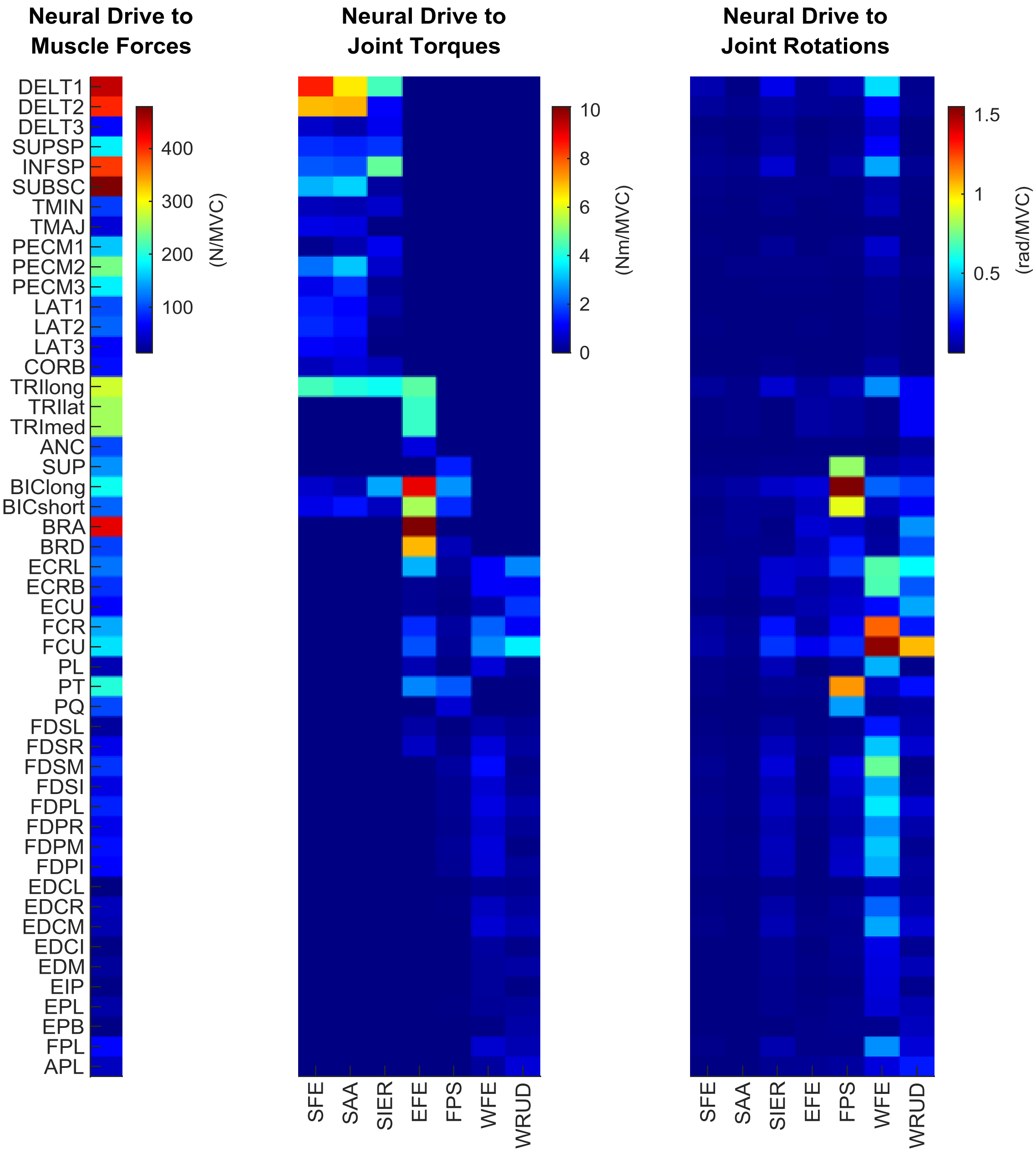

Figure SM3. Gains from neural drive to muscle force, joint torque, and joint displacement for 6 Hz tremor in Posture 3. See the caption to ***Error! Reference source not found.***1 for more details.

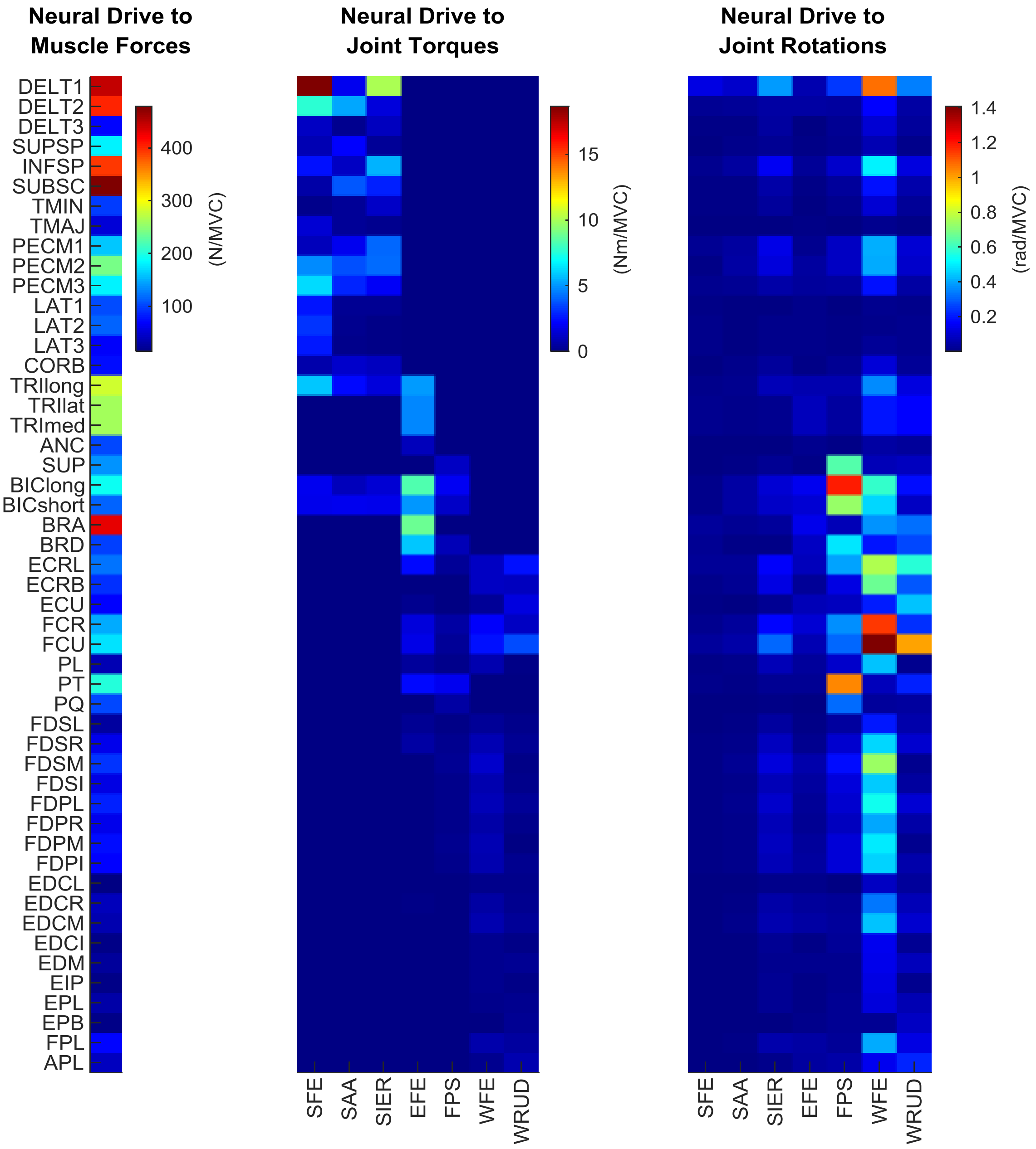

Figure SM4. Gains from neural drive to muscle force, joint torque, and joint displacement for 6 Hz tremor in Posture 4. See the caption to ***Error! Reference source not found.***1 for more details.

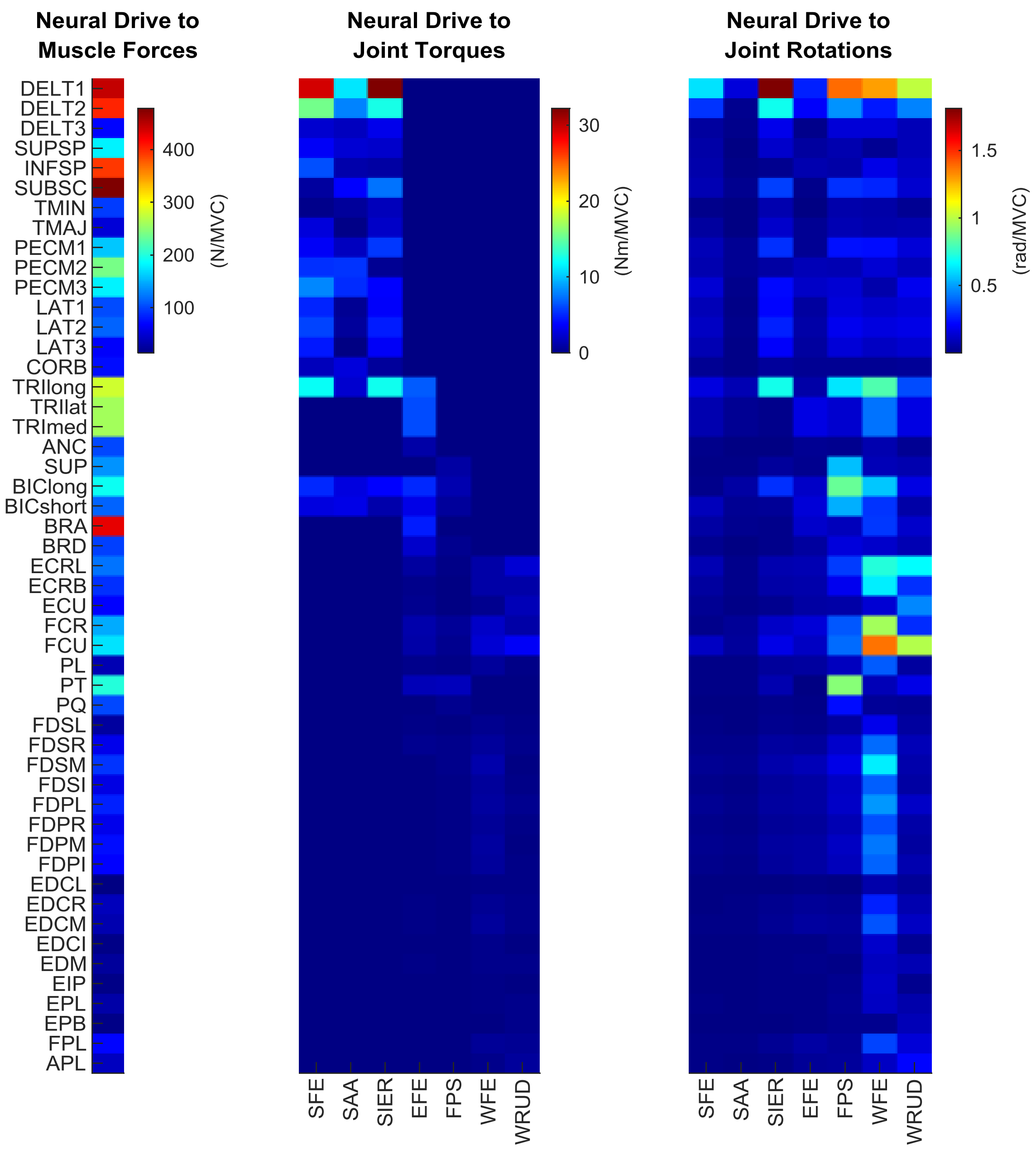

Figure SM5. Gains from neural drive to muscle force, joint torque, and joint displacement for 6 Hz tremor in Posture 5. See the caption to ***Error! Reference source not found.***1 for more details.

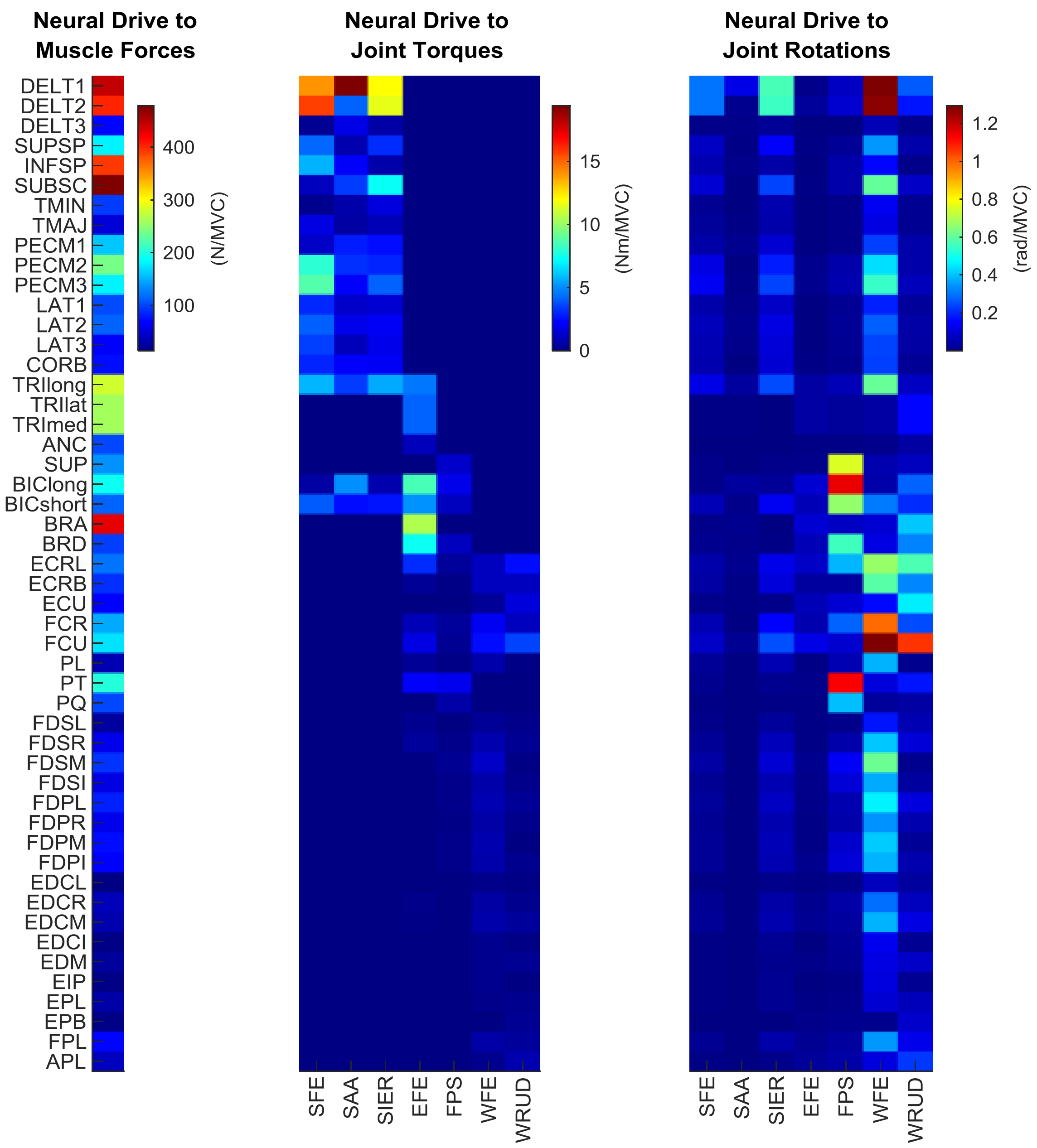

Figure SM6. Gains from neural drive to muscle force, joint torque, and joint displacement for 6 Hz tremor in Posture 6. See the caption to ***Error! Reference source not found.***1 for more details.

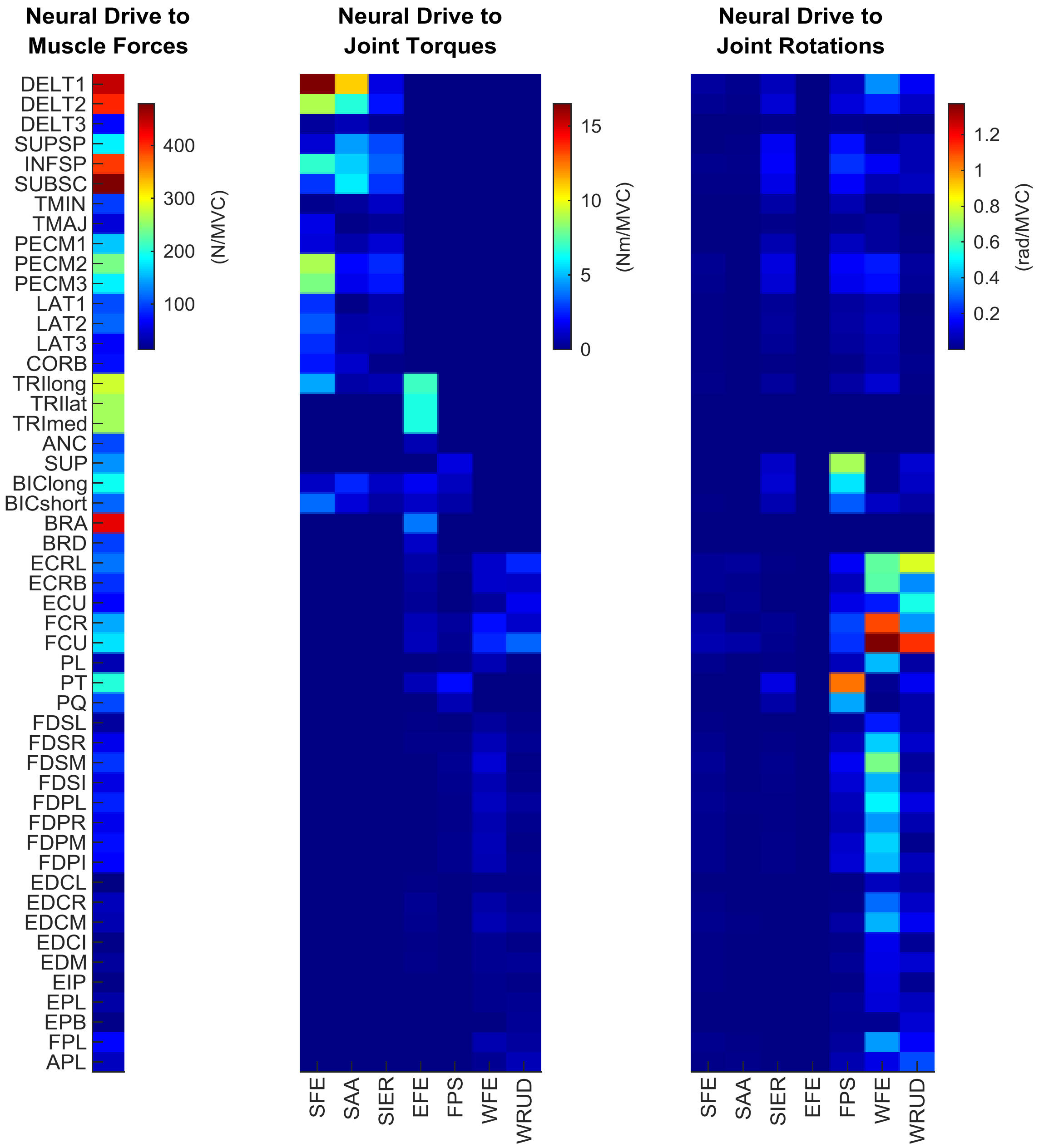

Figure SM7. Gains from neural drive to muscle force, joint torque, and joint displacement for 6 Hz tremor in Posture 7. See the caption to ***Error! Reference source not found.***1 for more details.

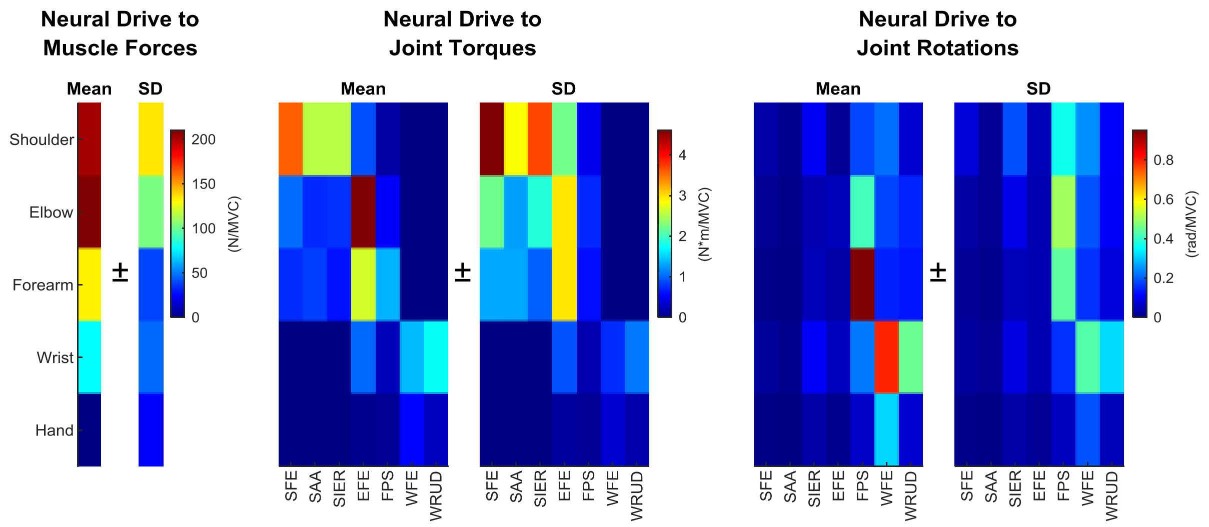

Figure SM8. Gains from neural drive to muscle force, neural drive to joint torque, and neural drive to joint rotation, averaged across all postures and muscles within each muscle group (evaluated with standard parameters at a tremor frequency of 6 Hz). For this analysis, the impact of a muscle was included at every joint it crossed (see Table 1), so shoulder muscles are defined as DELT1 to TRIlong plus BIClong and BICshort; elbow muscles as TRIlong to BRD; forearm muscles as SUP, BIClong, BICshort, PT, and PQ; wrist muscles are defined as ECRL to PL; and hand muscles are defined as FDSL to APL.

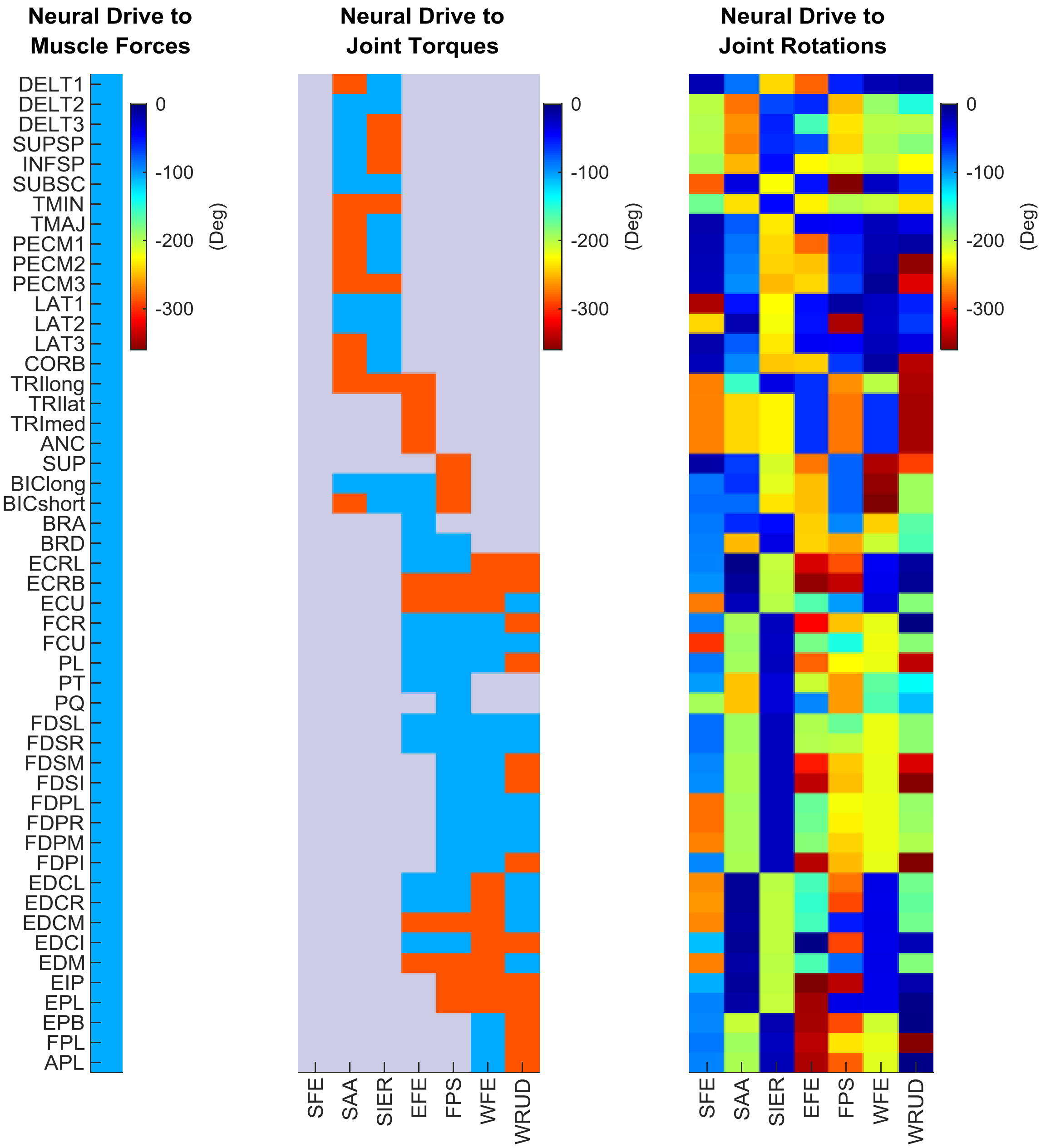

Figure SM9. Phase shifts of muscle force, joint torque, and joint displacement relative to neural drive (calculated for 6 Hz tremor in Posture 1). Phase shift values were calculated under the assumption of steady-state and were therefore shifted to lie between 0 and -360°. Grayed joint torques indicate torques with zero magnitude, so phase shifts are undefined. Muscles are ordered proximal (top) to distal (bottom; for muscle names, see Table 1 in the body of the article). Similarly, joint DOF are listed proximal (left) to distal (right): shoulder flexion-extension (SFE), abduction-adduction (SAA), and internal-external rotation (SIER); elbow flexion-extension (EFE) and forearm pronation-supination (FPS); and wrist flexion-extension (WFE) and radial-ulnar deviation (WRUD), respectively.

References

[1] A. D. Davidson and S. K. Charles, "Fundamental Principles of Tremor Propagation in the Upper Limb," *Annals of Biomedical Engineering*, vol. 45, no. 4, pp. 1133-1147, 2017, doi: 10.1007/s10439-016-1765-5.

[2] C. D. Fryar, M. D. Carroll, Q. Gu, J. Afful, and C. L. Ogden, "Anthropometric reference data for children and adults: United States, 2015–2018," *Vital and Health Statistics*, vol. 3, no. 46, pp. 7-15, 2021.

[3] P. De Leva, "Adjustments to Zatsiorsky-Seluyanov's segment inertia parameters," *J biomech*, vol. 29, no. 9, pp. 1223-1230, 1996.

[4] T. H. Corie and S. K. Charles, "Simulated Tremor Propagation in the Upper Limb: From Muscle Activity to Joint Displacement," *Journal of Biomechanical Engineering*, vol. 141, no. 8, p. 081001, 2019, doi: 10.1115/1.4043442.
